# Association of Genetic Risk for Rheumatoid Arthritis With Cognitive and Psychiatric Phenotypes Across Childhood and Adolescence

**DOI:** 10.1001/jamanetworkopen.2019.6118

**Published:** 2019-06-21

**Authors:** Hannah J. Jones, Leon Hubbard, Ruth E. Mitchell, Simon A. Jones, Nigel M. Williams, Stanley Zammit, Jeremy Hall

**Affiliations:** 1 MRC Integrative Epidemiology Unit at the University of Bristol, Bristol, United Kingdom; 2 Centre for Academic Mental Health, Population Health Sciences, Bristol Medical School, University of Bristol, Bristol, United Kingdom; 3 National Institute for Health Research Bristol Biomedical Research Centre, University Hospitals Bristol NHS Foundation Trust, University of Bristol, Bristol, United Kingdom; 4 Division of Psychological Medicine and Clinical Neurosciences, MRC Centre for Neuropsychiatric Genetics and Genomics, Cardiff University, Cardiff, United Kingdom; 5 Division of Infection and Immunity, School of Medicine, Systems Immunity University Research Institute, College of Biomedical and Life Sciences, Cardiff University, Cardiff, United Kingdom; 6 The Hodge Centre for Neuropsychiatric Immunology, Cardiff University, Cardiff, United Kingdom; 7 Neuroscience and Mental Health Research Institute, Cardiff University, Cardiff, United Kingdom

## Abstract

**Importance:**

The association of rheumatoid arthritis (RA) with cognitive and psychiatric phenotypes has been recognized. However, it is not known whether these phenotypes are a consequence of disease-related factors, such as pain, or reflect shared etiological factors.

**Objective:**

To investigate whether genomic risk for RA is associated with cognitive and psychiatric symptoms in children and adolescents.

**Design, Setting, and Participants:**

This cohort study analyzed data from 3296 to 5936 adolescents (depending on outcome) from the Avon Longitudinal Study of Parents and Children. Clinical and questionnaire data were collected periodically from September 6, 1990, with collection ongoing, and analyzed from August 21, 2017, to May 21, 2018.

**Exposures:**

Polygenic risk scores (PRSs) for RA.

**Main Outcomes and Measures:**

Measures of cognition (including IQ, working memory, verbal learning, processing speed, problem solving, selective attention, and attentional control) and psychopathology (including anxiety, depression, negative symptoms, psychotic experiences, attention-deficit/hyperactivity disorder, and hyperactive and inattentive symptoms) in childhood and adolescence.

**Results:**

Polygenic risk scores for RA were generated for 7977 children and adolescents (3885 [48.7%] female). Of these 7977 participants, 9 (0.11%) had a known diagnosis of RA at age 22 years. Increased PRS for RA was associated with lower total IQ (β, −0.05; 95% CI, −0.07 to −0.02; *P* < .001), performance IQ (β, −0.03; 95% CI, −0.06 to −0.005; *P* = .02), and verbal IQ (β, −0.05; 95% CI, −0.08 to −0.02; *P* < .001) at age 8 years (mean [SD] age at measurement, 8.6 [0.3] years) and symptoms of hyperactivity and inattention from ages 4 to 16 years, with the strongest evidence of association at age 13 years (mean [SD] age at assessment, 13.2 [0.2] years). The odds ratio at this age per SD increase in PRS was 1.25 (95% CI, 1.12-1.39) (*P* < .001). There was little evidence of association between the RA PRS and other measures of cognition and psychopathology. Gene-based analyses indicated that polygenic signal for RA was enriched for immune pathways (*q* ≤ 0.05). No equivalent associations were seen for polygenic risk associated with inflammatory bowel disease or multiple sclerosis.

**Conclusions and Relevance:**

These findings support an association between genetic risk for RA and neural phenotypes, suggesting that cognitive impairment in RA is not simply secondary to disease-related processes or treatment effects. These results may suggest that genetic susceptibility for RA might affect psychological well-being in early life and reinforce the emerging link between mental health and the immune system.

## Key Points

**Question:**

Is genetic liability for rheumatoid arthritis associated with cognitive and psychiatric phenotypes in children prior to the clinical onset of disease?

**Findings:**

In this cohort study of 7977 children and adolescents, genetic liability for rheumatoid arthritis was associated with lower total, performance, and verbal IQ at age 8 years and symptoms of hyperactivity and inattention from ages 4 to 16 years. However, there was little evidence of association with other domains of psychopathology.

**Meaning:**

These findings support a primary etiological association between genetic risk for rheumatoid arthritis and cognitive phenotypes that is not simply secondary to disease-related processes or reverse causation.

## Teaser

This cohort study investigates whether genomic risk for rheumatoid arthritis is associated with cognitive and psychiatric symptoms in children and adolescents.

## Introduction

Rheumatoid arthritis (RA) is a highly heritable chronic inflammatory disease that affects between 0.5% and 1% of the population and is most common in older adults.^1^ It has long been recognized that there are elevated rates of cognitive and psychiatric phenotypes in patients with RA.^2,3^ For example, a number of studies^4,5^ have reported evidence of specific areas of cognitive difficulty in adults with RA, including poorer performance in tests of verbal IQ, attention, and memory.^4^ Patients with RA are also reported to have increased rates of a number of psychiatric conditions.^6^ Rates of depression in individuals with RA are elevated to 2 to 4 times the levels seen in the general population.^2,3,7^ Similarly increased rates of anxiety disorders are also reported.^2^ While mental disorders such as depression and anxiety are more common in individuals with RA, the association between RA and psychotic disorders such as schizophrenia is more complex. Epidemiological studies have shown decreased rates of RA in those with schizophrenia.^8^ However, this has not been a universal finding, and uncertainty remains about whether there are altered rates of psychotic disorders in people with RA.^9^ There is also evidence that the offspring of mothers with RA are more likely to suffer from attention-deficit/hyperactivity disorder (ADHD), although it is not known whether this reflects shared genetic vulnerability across the disorders or exposure to the heightened inflammatory status (or associated medication) in the mother.^10^

Despite the robust evidence of cognitive deficits and increased presence of some psychiatric disorders in people with RA, the mechanism underlying these associations is unknown. Rheumatoid arthritis is not typically considered a disorder of the brain, but multiple disease-related factors could potentially affect central nervous system (CNS) function. These include the effects of RA symptoms such as pain; treatment with disease-modifying antirheumatic or biological drugs known to have CNS effects; the impact of heightened systemic inflammation on the brain; indirect effects of RA symptoms, such as decreased activity levels or social isolation; and a direct effect of shared risk factors for RA on brain function. The latter possibility is particularly intriguing given the increasing evidence that the immune system plays a direct role in nervous system development and function.^11,12,13^ Delineating the significance of each of these associations in patients with active disease is difficult owing to their health status and clinical management. However, general population studies examining the effects of risk factors for RA provide an opportunity to study alternative explanations for these associations while excluding RA disease-related mechanisms.

Recent large-scale meta-analyses of genome-wide association studies (GWAS) of RA have shown that there is a significant association of common genetic variants with disease risk.^14^ Using these data, we have used a polygenic risk score (PRS) approach^15,16,17^ to examine whether genetic liability for RA is associated with cognitive and psychiatric phenotypes in children and adolescents prior to the clinical onset of autoimmune disease and disease-related factors. To investigate the selectivity of our results to RA we also compared results with those obtained from associations between PRSs for 2 additional inflammatory disorders, inflammatory bowel disease (IBD) and multiple sclerosis (MS).

## Methods

### Study Population

The sample comprised young individuals within the Avon Longitudinal Study of Parents and Children (ALSPAC) longitudinal birth cohort (eMethods in the Supplement).^18,19^ The ALSPAC children have had detailed longitudinal assessments of health since birth, and details of all available data can be found through a fully searchable data dictionary and variable search tool.^20^ All ALSPAC study participants who complete questionnaires consent to the use of their data by approved researchers. Until age 18 years, an overarching parental consent was used to indicate parents consented for their child (the study participant) to take part in ALSPAC. Consent for data collection and use, implied via the written completion of questionnaires and parental consent, as well as assent from the child, was required for all physical measures and tissue sampling. Study participants have the right to withdraw their consent for specific elements of the study, or from the study as a whole, at any time. Ethical approval for the study was obtained from the ALSPAC Ethics and Law Committee and the local research ethics committees.

### Polygenic Risk Scores for Inflammatory Disorders

Genetic data were available for 7977 ALSPAC participants (eMethods in the Supplement). Polygenic risk scores were constructed using summary statistics from large GWASs for RA,^14^ IBD,^21^ and MS.^22^ The RA discovery sample contained 14 361 cases of RA and 43 923 controls, the IBD discovery sample contained 12 882 cases of Crohn disease or ulcerative colitis and 21 770 controls, and the MS discovery sample contained 9772 cases of MS and 17 376 controls. For all inflammatory disorders, single-nucleotide polymorphism (SNP) association data were taken from the GWAS limited to individuals of European decent.

Weighted PRSs were calculated for each ALSPAC individual using the PLINK genome association analysis software version 1.07 “score” command. Our primary analysis included PRSs generated using SNPs with an inflammatory disorder discovery sample association of *P* ≤ .05. Polygenic risk scores based on a range of GWAS *P* value thresholds (*P* ≤ .50 to *P* ≤ 1 × 10^−7^) were also generated and used in sensitivity analyses.

Owing to the substantial association between loci within the major histocompatibility complex (MHC) and inflammatory disorder risk, 2 versions of the PRSs were generated: 1 that included only a single SNP to represent the extended MHC region (chromosome 6: 25-34 Mb) and 1 that omitted all SNPs from the extended MHC region (eMethods in the Supplement).

### Cognitive Measures

Cognitive testing was carried out during a clinic visit of ALSPAC children at age 8 years using a short form of the Wechsler Intelligence Scale for Children, third edition^23^ and Test of Everyday Attention for Children.^24^ Measures captured using the Wechsler Intelligence Scale for Children included total IQ, performance IQ, verbal IQ, working memory, verbal learning, processing speed, and problem solving. Measures captured using the Test of Everyday Attention for Children included selective attention and attentional control. An additional measure of IQ was also captured at age 15 years during a clinic visit using the Wechsler Abbreviated Scale of Intelligence.^25^ All cognitive measures were standardized to have a mean of 0 and a standard deviation of 1 before analysis. The eMethods in the Supplement include additional details on all measures.

### Psychiatric Measures

Measures of psychopathology were taken at multiple ages (ranging from 4 to 18 years). Measures include presence of anxiety (at age 18 years), depression (at age 18 years) (both measured using the Clinical Interview Schedule-Revised^26^), negative symptoms (at age 16 years; measured using the Community Assessment of Psychic Experiences self-report questionnaire^27^), psychotic experiences (at ages 12 and 18 years; measured using the semistructured Psychosis-Like Symptom Interview^28,29^), ADHD (at age 7 years; measured using the Development and Well Being Assessment^30^), and hyperactive and inattentive symptoms (at ages 4, 7, 8, 10, 12, 13, and 16 years; measured using the parent-rated 5-item Strengths and Difficulties Questionnaire^31^).

### Inflammatory Markers

The proinflammatory cytokine interleukin 6 and the acute-phase protein C-reactive protein were measured using nonfasting blood samples collected in ALSPAC participants at age 9 years using methods described previously.^32^ C-reactive protein was also measured using fasting blood samples collected in ALSPAC participants at age 16 years using methods described previously.^33^ Inflammatory marker measures were log transformed before use.

### Gene-Based Analyses

To investigate the molecular pathways associated with genetic risk for RA, and to determine whether any of these contribute to the observed associations with cognitive and psychiatric phenotypes, gene-based statistics were derived using results of the RA GWAS^14^ using Multi-marker Analysis of GenoMic Annotation,^34^ which was also used to test whether RA genes were enriched in 7321 gene-ontology gene sets (excluding genes within the extended MHC region) (eMethods in the Supplement).

For gene sets surviving multiple testing correction, hierarchical clustering analysis was used to identify gene sets that were correlated based on shared gene overlap as implemented in the package “stats” in R statistical software version 3.3.1 (R Project for Statistical Computing). Results of the hierarchical clustering analysis were plotted in as a dendrogram format and broader cluster definitions were derived via manual inspection (eFigure 1 in the Supplement). Polygenic risk scores for RA based on SNPs within each gene set cluster were then derived using the methods previously mentioned.

### Statistical Analysis

Univariate linear regression was used to test associations between inflammatory disorder PRSs and continuous measures, including all cognitive measures and inflammatory markers. Univariate logistic regression was used to test associations between inflammatory disorder PRSs and dichotomous measures, including presence of anxiety, depression, negative symptoms, psychotic experiences, ADHD, and hyperactive and inattentive symptoms. Results of linear regression analyses are presented as β coefficients (standardized unit changes in the dependent variable per standardized unit change in the inflammatory disorder PRS) and results of logistic regression analyses are presented as odds ratios (odds that the dependent variable will occur per standardized unit change in the inflammatory disorder PRS). A 2-tailed *P* value less than .05 was considered statistically significant. However, effect sizes and confidence intervals are presented to allow readers to gauge the range of values compatible with the true value in the population. All statistical analyses were performed in Stata statistical software version 15.1 (StataCorp LLC).

## Results

Polygenic risk scores for RA, IBD, and MS were derived for 7977 participants (3885 [48.7%] female) from the ALSPAC longitudinal birth cohort. Mean ages, analysis sample sizes, and descriptive statistics for each outcome are shown in eTables 1, 2, and 3 in the Supplement. Of the 7977 participants with PRS data, 9 (0.11%; 7 female and 2 male) had a known diagnosis of RA, 23 (0.29%; 15 female and 8 male) had a known diagnosis of IBD, and 1 (0.01%; female) had a known diagnosis of MS at age 22 years.

### Associations Between Polygenic Risk for RA and Cognitive Phenotypes

**Figure 1.**
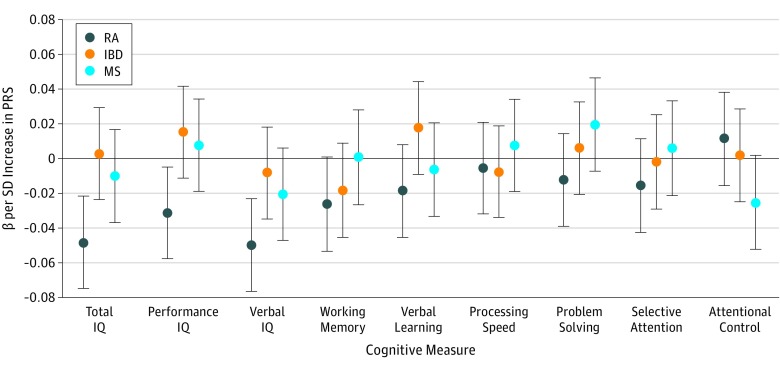
Associations Between Polygenic Risk Scores (PRSs) for Rheumatoid Arthritis (RA), Inflammatory Bowel Disease (IBD), and Multiple Sclerosis (MS) and Cognitive Measures at Age 8 Years Points represent β values, with error bars indicating 95% confidence intervals. β values are shown for PRSs generated using lists of single-nucleotide polymorphisms meeting a *P* value threshold of .05. The horizontal black line indicates the null value. The number of observations for total IQ was 5305; performance IQ, 5320; verbal IQ, 5328; working memory, 5210; verbal learning, 5334; processing speed, 5340; problem solving, 5282; selective attention, 5105; and attentional control, 5177. Numerical results used to generate the figure are presented in eTable 4 in the Supplement.

At age 8 years (mean [SD] age at measurement, 8.6 [0.3] years), the RA PRS was associated with a lower total IQ (β, −0.05; 95% CI, −0.07 to −0.02; *P* < .001; *R*^2^ = 0.002) (Figure 1; eTable 4 in the Supplement). Particularly strong findings were observed for verbal IQ (β, −0.05; 95% CI, −0.08 to −0.02; *P* < .001; *R*^2^ = 0.003) (Figure 1; eTable 4 in the Supplement), while evidence was weaker for performance IQ (β, −0.03; 95% CI, −0.06 to −0.005; *P* = .02; *R*^2^ = 0.001) (Figure 1; eTable 4 in the Supplement). There was little evidence of association between the RA PRS and other cognitive measures as collected by ALSPAC (Figure 1; eTable 4 in the Supplement).

To investigate the selectivity of our results to RA we further investigated associations between IBD and MS PRSs and measures of IQ and cognition (Figure 1). In contrast to the RA PRS, there was little evidence of association between the IBD or MS PRSs and IQ (Figure 1; eTable 4 in the Supplement).

### Associations Between Polygenic Risk for RA and Psychiatric Phenotypes

**Figure 2.**
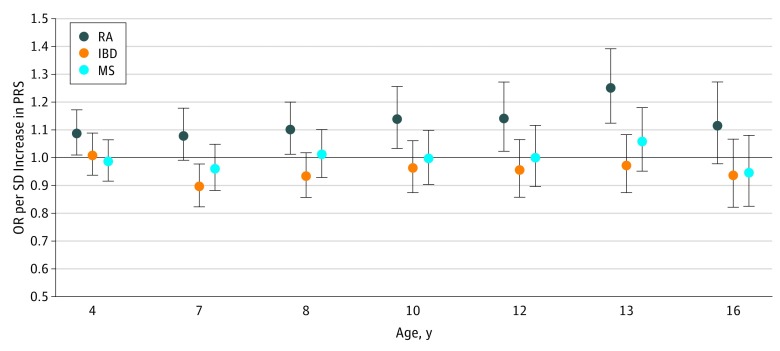
Associations Between Polygenic Risk Scores (PRSs) for Rheumatoid Arthritis (RA), Inflammatory Bowel Disease (IBD), and Multiple Sclerosis (MS) and Hyperactive and Inattentive Symptoms Measures at Multiple Ages Points represent odds ratios (ORs), with error bars indicating 95% confidence intervals. Odds ratios are shown for PRSs generated using lists of single-nucleotide polymorphisms meeting a *P* value threshold of .05. The horizontal black line indicates the null value. The number of observations for age 4 years was 5936; 7 years, 5531; 8 years, 5291; 10 years, 5551; 12 years, 5129; 13 years, 4953; and 16 years, 4089. Numerical results used to generate the figure are presented in eTable 5 in the Supplement.

Polygenic risk scores for RA were associated with increased odds of hyperactive and inattentive symptoms measured between approximate ages of 4 and 16 years. The strongest evidence of association was at age 13 years (mean [SD] age at assessment, 13.2 [0.2] years) (odds ratio, 1.25; 95% CI, 1.12-1.39; *P* < .001; pseudo *R*^2^ = 0.007) (Figure 2; eTable 5 in the Supplement).

There was little consistent evidence of association between RA, IBD, or MS PRSs and anxiety or depression at age 18 years, negative symptoms at age 16 years, psychotic experiences at age 12 or 18 years, or Development and Well Being Assessment bands predicting a 15% or greater probability of clinical diagnosis of ADHD at age 7 years (eFigure 2 and eTable 5 in the Supplement).

### Associations Between Polygenic Risk for RA and Inflammatory Markers

**Figure 3.**
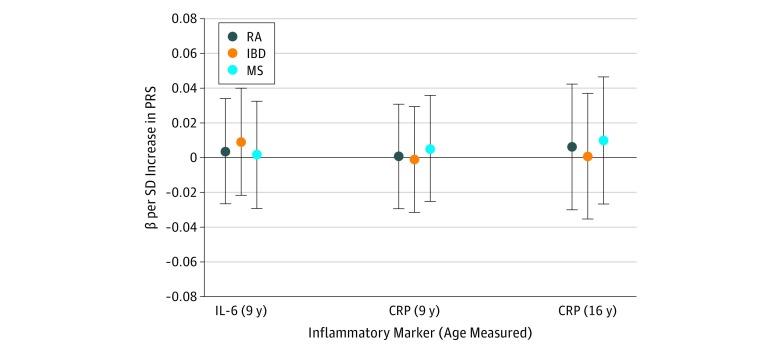
Associations Between Polygenic Risk Scores (PRSs) for Rheumatoid Arthritis (RA), Inflammatory Bowel Disease (IBD), and Multiple Sclerosis (MS) and Inflammatory Markers Points represent β values for interleukin 6 (IL-6) and C-reactive protein (CRP), with error bars indicating 95% confidence intervals. β values are shown for PRSs generated using lists of single-nucleotide polymorphisms meeting a *P* value threshold of .05. The horizontal black line indicates the null value. The number of observations for IL-6 at age 9 years was 4055; CRP at age 9 years, 4064; and CRP at age 16 years, 2779. Numerical results used to generate the figure are presented in eTable 6 in the Supplement.

There was no association between RA PRSs and plasma interleukin 6 (β, 0.004; 95% CI, −0.03 to 0.03; *P* = .81; *R*^2^ < 0.001) or plasma C-reactive protein (β, 0.001; 95% CI, −0.03 to 0.03; *P* = .96; *R*^2^ < 0.001) at age 9 years (mean [SD] age at measurement, 9.9 [0.3] years). There was also no association between RA PRSs and plasma C-reactive protein at age 16 years (mean [SD] age at measurement, 15.5 [0.4] years) (β, 0.01; 95% CI, −0.03 to 0.04; *P* = .74; *R*^2^ < 0.001) or between IBD or MS PRSs and any inflammatory marker (Figure 3; eTable 6 in the Supplement).

### Gene-Based Analyses of RA SNPs

After correcting for multiple testing, 106 gene sets were associated with RA (*q* ≤ 0.05), extending the previously reported pathway analysis of this GWAS.^14^ Using the proportion of genes overlapping each of the 106 gene sets and their biological descriptions, we derived 2 gene set clusters representing biological similarity of individually significant gene sets and 9 gene set subclusters. The subclusters of gene set cluster 1 were defined as lymphocyte activation and lymphocyte differentiation. The subclusters of gene set cluster 2 were defined as activated lymphocyte homing, lymphocyte effector functions, immune activation, Th2 effector characteristics, Th1 and Th17 effector characteristics, lymphokine activities, and immune effector functions (eFigure 1 in the Supplement).

Polygenic risk scores for RA based on SNPs within each gene set subcluster and SNPs within clusters 1 and 2, separately and combined, showed little evidence of association with IQ at age 8 years or hyperactive and inattentive symptoms at age 13 years (eFigure 3, eFigure 4, eTable 7, and eTable 8 in the Supplement).

### Sensitivity Analyses

The evidence of association between RA PRSs and IQ measures at age 8 years and hyperactive and inattentive symptoms was similar when using PRSs omitting all SNPs from the extended MHC region and after excluding the 9 individuals who reported an RA diagnosis at age 22 years and individuals whose mothers had been told by a doctor that they have arthritis (eFigure 5, eFigure 6, eTable 9, and eTable 10 in the Supplement). Results were also similar when using RA PRSs based on *P* value thresholds ranging from less than or equal to .50 to less than or equal to 1 × 10^−3^ for IQ (eFigure 7 and eTable 11 in the Supplement) and *P* value thresholds ranging from less than or equal to .50 to less than or equal to .01 for hyperactive and inattentive symptoms (eFigure 8 and eTable 12 in the Supplement). An association between RA PRSs and IQ (β, −0.04; 95% CI, −0.07 to −0.01; *P* = .02; *R*^2^ = 0.001; N = 3961), but not between IBD PRSs and IQ (β, −0.01; 95% CI, −0.04 to 0.03; *P* = .71; *R*^2^ < 0.0001; N = 3961) or MS PRSs and IQ (β, −0.02; 95% CI, −0.05 to 0.01; *P* = .28; *R*^2^ = 0.0003; N = 3961), was also evident at age 15 years.

## Discussion

In this study, we have shown that within a general population development cohort, polygenic risk for RA was associated with measures of IQ at age 8 years and hyperactivity and inattentive symptoms (most strongly at age 13 years). This supports a primary etiological association between genetic risk for RA and neural phenotypes that is not simply secondary to disease-related processes or reverse causation.

We found an association between polygenic risk for RA and IQ, with stronger evidence of association between RA PRS and verbal IQ as compared with performance IQ. This finding is notable because studies in patient groups have shown that verbal function is one of the most robustly affected areas of cognition in RA.^4^ Previous studies have also suggested an association between RA risk and later cognitive impairment and dementia.^35,36^ The present results suggest that some of this vulnerability may be mediated through the effects of genetic risk for RA on cognitive function and may not solely be caused by disease-related effects or medication.

We also found that the RA PRS was associated with increased scores on measures of inattention and hyperactivity during childhood. This association was particularly clear across developmental points associated with the presentation of ADHD. Our results are consistent with a recent large population study that showed an increased incidence of ADHD in mothers with RA.^10^ The present findings suggest that this association may result from shared genetic risk factors, rather than from other factors such as the effects of disease-related inflammation during fetal development, as the mothers of children in the current study did not predominantly report having RA or related disorders, and our results remained robust even after excluding those who did report such symptoms. Our results are also broadly in keeping with the finding that attention is a domain of cognition particularly affected in patients with RA,^4^ although the current findings suggest a particularly strong association of genetic risk for RA with attention during childhood development.

We observed little evidence of association between RA PRS and depression or anxiety-related phenotypes in this developmental cohort, even though these symptom domains have been previously associated with RA. This may reflect prior evidence that these phenotypes are particularly influenced by disease-associated and social factors (including disease severity, pain, and lower socioeconomic status).^3^ In fact, while rates of depression are high in patients with RA, they are typical of the levels seen in a range of chronic diseases.^2,37^ It is also possible that PRS for RA may be associated with risk for these traits, but at a later developmental point as phenotypes such as depression most often emerge in later adolescent and adult life. We also did not detect an association between RA PRS and psychotic symptoms in these younger individuals, unlike some previous studies reporting a small overlap between genetic risk for RA and schizophrenia,^38^ although results from prior studies have not been consistent.^39,40^

Our pathway analysis confirms that, even after excluding MHC loci, PRS for RA was associated with effects on immune pathways. The finding that RA PRSs were associated with cognitive and psychiatric phenotypes therefore supports the emerging view that immune pathways play an important role in brain development and function.^11,13^ Furthermore, in our healthy population, the associations were seen in the absence of inflammatory disease or elevated peripheral immune markers, suggesting a direct CNS effect. Notably, recent studies have begun to demonstrate direct effects of specific immune pathways, including the complement system and T-cell activation, in neurodevelopment and behavioral phenotypes.^12,41^ An important future direction will, therefore, be to dissect the specific contribution of components of the immune system to nervous system function.

In contrast to the RA findings, we found little evidence of association between the IBD or MS PRSs and cognitive or psychiatric symptoms in childhood and adolescence. Previous studies have suggested increased rates of depression in IBD.^42,43^ However, akin to RA, these rates are in line with those seen in chronic diseases more generally. Although there is currently little evidence of specific cognitive impairments associated with IBD, cognitive impairments in MS are well documented.^44^ Our findings suggest that the latter potentially occur secondary to illness onset rather than occurring as earlier (premorbid) changes as observed for RA. However, the MS GWAS had the smallest sample size of all inflammatory disorders and, therefore, analyses using the MS PRSs may be underpowered compared with those for IBD and RA, whose PRSs were based on GWAS samples of similar, and larger, magnitude. It is also important to note that the IBD GWAS analyses combine data both from patients with ulcerative colitis and Crohn disease. While these diseases are clearly closely related, it is possible that future studies may identify a stronger association of genetic risk for one of these conditions alone with neuropsychiatric phenotypes. Overall, the lack of association between PRSs for IBD or MS and cognitive and psychiatric outcomes in the current study suggests a specific effect of pathways associated with genetic risk for RA on the CNS.

### Strengths and Limitations

A strength of our study is that we used a large, well-characterized longitudinal cohort, albeit of mostly European ancestry, with multiple self-report– and interview-based measures on cognition and psychopathology collected prospectively. However, despite the size of our study, power may have limited our ability to observe associations with uncommon outcomes. This could explain why we observed evidence of an association between RA genetic risk and attention and hyperactivity but did not find an association with syndromal ADHD (based on the top 2 Development and Well Being Assessment bands,^45^ estimated prevalence of ADHD at age 7 years was 2.53%). Further analyses in larger sample sizes are needed to investigate whether genetic liability for RA is associated with ADHD as well as subclinical difficulties.

We also cannot account for potential parental RA effects on child outcomes that are not mediated through the child’s genetic risk but that are mediated, for example, through effects on the child’s upbringing. However, excluding individuals whose mothers reported an arthritis diagnosis had little effect on the results, and while we did not have data on paternal RA, such an explanation seems unlikely given the mean age at onset of RA^46^ and the ages of child phenotypes studied.

As with most longitudinal cohorts, attrition in ALSPAC leads to underrepresentation of lower social classes.^28^ Genetic liability for multiple lifestyle factors, personal characteristics, and health conditions has also been shown to influence participation in ALSPAC.^47^ Therefore, our estimates for IQ and attention and hyperactivity might be affected by selection bias, although one might expect such a bias to affect all the cognitive and psychiatric phenotypes investigated within this study to a similar degree.

## Conclusions

The associations observed within the current study have implications in terms of the clinical assessment and management of RA. Overall, RA is currently conceptualized as a multisystem connective tissue disorder. The current results, however, suggest that CNS impacts should also be considered a core and primary component of RA, especially in the domain of cognition. Furthermore, they support the view that clinically it is important to assess and monitor cognitive impairment in patients with RA, especially as cognitive factors can affect activities of daily living and individuals’ overall level of functioning.^4^ Further studies of the role of immune pathways in regulating cognitive function may also lead to the development of new therapeutic approaches for cognitive impairments in RA and, more broadly, for areas such as ADHD.

## Notes

Supplement. **eMethods. **Additional Information on Study Population, Measures, and Analyses **eReferences** **eFigure 1.** Identified Gene-Sets and Gene-Set Sub-clusters **eFigure 2.** Associations Between Polygenic Risk Scores (PRSs) for Rheumatoid Arthritis (RA), Inflammatory Bowel Disease (IBD) and Multiple Sclerosis (MS), and Psychopathology Measures **eFigure 3.** Associations Between Polygenic Risk Scores (PRSs) for Rheumatoid Arthritis Based on Single-Nucleotide Polymorphisms (SNPs) Within Identified Gene-Set Clusters, and IQ at Age 8 Years **eFigure 4.** Associations Between Polygenic Risk Scores (PRSs) for Rheumatoid Arthritis Based on Single-Nucleotide Polymorphisms (SNPs) Within Identified Gene-Set Clusters, and Hyperactive and Inattentive Symptoms at Age 13 Years **eFigure 5.** Sensitivity Analysis for Associations Between Rheumatoid Arthritis (RA) Polygenic Risk Scores (PRSs) and Cognitive Phenotypes at Age 8 Years **eFigure 6.** Sensitivity Analysis for Associations Between Rheumatoid Arthritis (RA) Polygenic Risk Scores (PRSs) and Hyperactive and Inattentive Symptoms Measures at Multiple Ages **eFigure 7.** Associations Between Polygenic Risk Scores (PRSs) for Rheumatoid Arthritis (RA), Inflammatory Bowel Disease (IBD) and Multiple Sclerosis (MS), and IQ Phenotypes at Age 8 Years **eFigure 8.** Associations Between Polygenic Risk Scores (PRSs) for Rheumatoid Arthritis (RA), Inflammatory Bowel Disease (IBD) and Multiple Sclerosis (MS), and Hyperactive and Inattentive Symptoms at Age 13 Years **eTable 1.** Mean Age, Number of Individuals and Mean Measure for Each Cognitive Outcome Measure for the Total Sample and Sample With Genetic Data **eTable 2.** Mean Age, Number of Individuals and Proportion of Sample With Psychopathology for Each Psychopathology Outcome Measure for the Total Sample and Sample With Genetic Data **eTable 3.** Mean Age, Number of Individuals and Mean Level of Each Inflammatory Marker Measure for the Total Sample and Sample With Genetic Data **eTable 4.** Associations Between Cognitive Measures at Age 8 Years and Polygenic Risk Scores for Inflammatory Disorders Generated Using Lists of Single-Nucleotide Polymorphisms Meeting a *P*-value Threshold of 0.05 **eTable 5.** Associations Between Measures of Psychopathology and Polygenic Risk Scores for Inflammatory Disorders Generated Using Lists of Single-Nucleotide Polymorphisms Meeting a *P*-value Threshold of 0.05 **eTable 6.** Associations Between Measures of Inflammatory Markers and Polygenic Risk Scores for Inflammatory Disorders Generated Using Lists of Single-Nucleotide Polymorphisms Meeting a *P*-Value Threshold of 0.05 **eTable 7.** Associations Between Polygenic Risk Scores for Rheumatoid Arthritis Based on Single-Nucleotide Polymorphisms Meeting a *P*-value Threshold of 0.05 Within Identified Gene-Set Clusters, and IQ at Age 8 Years **eTable 8.** Associations Between Polygenic Risk Scores for Rheumatoid Arthritis Based on Single-Nucleotide Polymorphisms Meeting a *P*-value Threshold of 0.05 Within Identified Gene-Set Clusters, and Hyperactive and Inattentive Symptoms at Age 13 Years **eTable 9.** Sensitivity Analysis for Associations Between Rheumatoid Arthritis Polygenic Risk Scores and Cognitive Phenotypes at Age 8 Years **eTable 10.** Sensitivity Analysis for Associations Between Rheumatoid Arthritis Polygenic Risk Scores and Hyperactive and Inattentive Symptoms Measures at Multiple Ages **eTable 11.** Associations Between Polygenic Risk Scores for Rheumatoid Arthritis, Inflammatory Bowel Disease and Multiple Sclerosis, and IQ Phenotypes at Age 8 Years Across a Range of Polygenic Risk Score *P* Value Thresholds **eTable 12.** Associations Between Polygenic Risk Scores for Rheumatoid Arthritis, Inflammatory Bowel Disease and Multiple Sclerosis, and Hyperactive and Inattentive Symptoms at Age 13 Years Across a Range of Polygenic Risk Score *P* Value Thresholds
