# Supplementary material for: Association of Genetic Risk for Rheumatoid Arthritis With Cognitive and Psychiatric Phenotypes Across Childhood and Adolescence
Source: JAMA Netw Open. 2019 Jun 21;2(6):e196118. doi: 10.1001/jamanetworkopen.2019.6118 (PMC6593640; doi:10.1001/jamanetworkopen.2019.6118)
Supplement: Supplement. — eMethods. Additional Information on Study Population, Measures, and Analyses eReferences eFigure 1. Identified Gene-Sets and Gene-Set Sub-clusters eFigure 2. Associations Between Polygenic Risk Scores (PRSs) for Rheumatoid Arthritis (RA), Inflammatory Bowel Disease (IBD) and Multiple Sclerosis (MS), and Psychopathology Measures eFigure 3. Associations Between Polygenic Risk Scores (PRSs) for Rheumatoid Arthritis Based on Single-Nucleotide Polymorphisms (SNPs) Within Identified Gene-Set Clusters, and IQ at Age 8 Years eFigure 4. Associations Between Polygenic Risk Scores (PRSs) for Rheumatoid Arthritis Based on Single-Nucleotide Polymorphisms (SNPs) Within Identified Gene-Set Clusters, and Hyperactive and Inattentive Symptoms at Age 13 Years eFigure 5. Sensitivity Analysis for Associations Between Rheumatoid Arthritis (RA) Polygenic Risk Scores (PRSs) and Cognitive Phenotypes at Age 8 Years eFigure 6. Sensitivity Analysis for Associations Between Rheumatoid Arthritis (RA) Polygenic Risk Scores (PRSs) and Hyperactive and Inattentive Symptoms Measures at Multiple Ages eFigure 7. Associations Between Polygenic Risk Scores (PRSs) for Rheumatoid Arthritis (RA), Inflammatory Bowel Disease (IBD) and Multiple Sclerosis (MS), and IQ Phenotypes at Age 8 Years eFigure 8. Associations Between Polygenic Risk Scores (PRSs) for Rheumatoid Arthritis (RA), Inflammatory Bowel Disease (IBD) and Multiple Sclerosis (MS), and Hyperactive and Inattentive Symptoms at Age 13 Years eTable 1. Mean Age, Number of Individuals and Mean Measure for Each Cognitive Outcome Measure for the Total Sample and Sample With Genetic Data eTable 2. Mean Age, Number of Individuals and Proportion of Sample With Psychopathology for Each Psychopathology Outcome Measure for the Total Sample and Sample With Genetic Data eTable 3. Mean Age, Number of Individuals and Mean Level of Each Inflammatory Marker Measure for the Total Sample and Sample With Genetic Data eTable 4. Associations Between Cognitive Measures at A [file jamanetwopen-2-e196118-s001.pdf]

Supplementary Online Content

Jones HJ, Hubbard L, Mitchell RE, et al. Association of genetic risk for rheumatoid arthritis with cognitive and psychiatric phenotypes across childhood and adolescence. *JAMA Netw Open*. 2019;2(6):e196118. doi:10.1001/jamanetworkopen.2019.6118

eMethods. Additional Information on Study Population, Measures, and Analyses

eReferences

eFigure 1. Identified Gene-Sets and Gene-Set Sub-clusters

eFigure 2. Associations Between Polygenic Risk Scores (PRSs) for Rheumatoid Arthritis (RA), Inflammatory Bowel Disease (IBD) and Multiple Sclerosis (MS), and Psychopathology Measures

eFigure 3. Associations Between Polygenic Risk Scores (PRSs) for Rheumatoid Arthritis Based on Single-Nucleotide Polymorphisms (SNPs) Within Identified Gene-Set Clusters, and IQ at Age 8 Years

eFigure 4. Associations Between Polygenic Risk Scores (PRSs) for Rheumatoid Arthritis Based on Single-Nucleotide Polymorphisms (SNPs) Within Identified Gene-Set Clusters, and Hyperactive and Inattentive Symptoms at Age 13 Years

eFigure 5. Sensitivity Analysis for Associations Between Rheumatoid Arthritis (RA) Polygenic Risk Scores (PRSs) and Cognitive Phenotypes at Age 8 Years

eFigure 6. Sensitivity Analysis for Associations Between Rheumatoid Arthritis (RA) Polygenic Risk Scores (PRSs) and Hyperactive and Inattentive Symptoms Measures at Multiple Ages

eFigure 7. Associations Between Polygenic Risk Scores (PRSs) for Rheumatoid Arthritis (RA), Inflammatory Bowel Disease (IBD) and Multiple Sclerosis (MS), and IQ Phenotypes at Age 8 Years

eFigure 8. Associations Between Polygenic Risk Scores (PRSs) for Rheumatoid Arthritis (RA), Inflammatory Bowel Disease (IBD) and Multiple Sclerosis (MS), and Hyperactive and Inattentive Symptoms at Age 13 Years

eTable 1. Mean Age, Number of Individuals and Mean Measure for Each Cognitive Outcome Measure for the Total Sample and Sample With Genetic Data

eTable 2. Mean Age, Number of Individuals and Proportion of Sample With Psychopathology for Each Psychopathology Outcome Measure for the Total Sample and Sample With Genetic Data

eTable 3. Mean Age, Number of Individuals and Mean Level of Each Inflammatory Marker Measure for the Total Sample and Sample With Genetic Data

© 2019 Jones HJ et al. *JAMA Network Open.*

eTable 4. Associations Between Cognitive Measures at Age 8 Years and Polygenic Risk Scores for Inflammatory Disorders Generated Using Lists of Single-Nucleotide Polymorphisms Meeting a *P*-value Threshold of 0.05

eTable 5. Associations Between Measures of Psychopathology and Polygenic Risk Scores for Inflammatory Disorders Generated Using Lists of Single-Nucleotide Polymorphisms Meeting a *P*-value Threshold of 0.05

eTable 6. Associations Between Measures of Inflammatory Markers and Polygenic Risk Scores for Inflammatory Disorders Generated Using Lists of Single-Nucleotide Polymorphisms Meeting a *P*-Value Threshold of 0.05

eTable 7. Associations Between Polygenic Risk Scores for Rheumatoid Arthritis Based on Single-Nucleotide Polymorphisms Meeting a *P*-value Threshold of 0.05 Within Identified Gene-Set Clusters, and IQ at Age 8 Years

eTable 8. Associations Between Polygenic Risk Scores for Rheumatoid Arthritis Based on Single-Nucleotide Polymorphisms Meeting a *P*-value Threshold of 0.05 Within Identified Gene-Set Clusters, and Hyperactive and Inattentive Symptoms at Age 13 Years

eTable 9. Sensitivity Analysis for Associations Between Rheumatoid Arthritis Polygenic Risk Scores and Cognitive Phenotypes at Age 8 Years

eTable 10. Sensitivity Analysis for Associations Between Rheumatoid Arthritis Polygenic Risk Scores and Hyperactive and Inattentive Symptoms Measures at Multiple Ages

eTable 11. Associations Between Polygenic Risk Scores for Rheumatoid Arthritis, Inflammatory Bowel Disease and Multiple Sclerosis, and IQ Phenotypes at Age 8 Years Across a Range of Polygenic Risk Score *P* Value Thresholds

eTable 12. Associations Between Polygenic Risk Scores for Rheumatoid Arthritis, Inflammatory Bowel Disease and Multiple Sclerosis, and Hyperactive and Inattentive Symptoms at Age 13 Years Across a Range of Polygenic Risk Score *P* Value Thresholds

This supplementary material has been provided by the authors to give readers additional information about their work.

© 2019 Jones HJ et al. *JAMA Network Open.*

eMethods. Additional information on study population, measures, and analyses

Study population

The sample comprised of young individuals within the ALSPAC longitudinal birth cohort. The initial cohort consisted of 14,541 pregnant women residing in the former Avon Health Authority area with an expected delivery date between April 1991 and December 1992.[1,2] Of these initial pregnancies, there was a total of 14,676 fetuses, resulting in 14,062 live births and 13,988 children who were alive at 1 year of age. When the oldest children were approximately 7 years of age, an attempt was made to bolster the initial sample with eligible cases who had failed to join the study originally. The total sample size for analyses using any data collected after the age of seven is therefore 15,247 pregnancies, resulting in 15,458 fetuses. Of this total sample of 15,458 fetuses, 14,775 were live births and 14,701 were alive at 1 year of age. ALSPAC study participants who complete questionnaires consent to the use of their data by approved researchers. Up until age 18 years an overarching parental consent was used to indicate parents were happy for their child (the study participant) to take part in ALSPAC. Consent for data collection and use was implied via the written completion of questionnaires and parental consent, as well as assent from the child, was required for all physical measures and tissue sampling. Study participants have the right to withdraw their consent for specific elements of the study, or from the study as a whole, at any time. Ethical approval for the study was obtained from the ALSPAC Ethics and Law Committee and the Local Research Ethics Committees.

Genetic data

Genetic data were acquired using the Illumina HumanHap550 quad genome-wide single-nucleotide polymorphism (SNP) genotyping platform from 9912 participants. Individuals were excluded from further analysis based on gender mismatches, minimal or excessive heterozygosity, disproportionate levels of individual missingness (>3%), evidence of cryptic relatedness (>10% of alleles identical by descent) and being of non-European ancestry (assessed by multidimensional scaling analysis including HapMap 2 individuals). SNPs with a minor allele frequency (MAF) of < 1%, Impute2 information quality metric of < 0.8, a call rate of < 95% or evidence for violations of Hardy-Weinberg equilibrium (p value < 5×10^{-7}) were removed. Imputation of the target data was performed using Impute V2.2.2 against the 1000 genomes reference panel (Phase 1, Version 3; all polymorphic SNPs excluding singletons), using 2186 reference haplotypes (including non-Europeans). Following quality control assessment and imputation and restricting to 1 young person per family, genetic data was available for 7977 ALSPAC individuals.

Polygenic risk scores for inflammatory disorders

Prior to construction of polygenic risk scores (PRSs), SNPs were removed from the analysis if they had a minor allele frequency less than 0.01, an imputation quality less than 0.8 or if there was allelic mismatch between samples (the alleles reported by the discovery study did not match the alleles present in the ALSPAC sample). Remaining SNPs were then further pruned for linkage disequilibrium (LD) using the PLINK (v1.90)[3] 'clump' command to retain SNPs with an inflammatory disorder association p value ≤ 0.5 and $r^2 < 0.25$ within 500kb windows.

PRSs were constructed using publicly available summary statistics of SNP associations from large genome-wide association studies (GWASs) for rheumatoid arthritis (RA),[4] inflammatory bowel disease (IBD)[5] and multiple sclerosis (MS).[6] For all inflammatory disorders (RA, IBD and MS), SNP association data were taken from the GWAS limited to individuals of European decent.

PRSs were calculated for each ALSPAC individual using the PLINK (v1.07) 'score' command. Scores are calculated by summing the number of reference alleles present for each SNP (0, 1 or 2) weighted by the logarithm of its odds ratio for each inflammatory disorder and standardized prior to analyses.

Due to the substantial link between loci within the major histocompatibility complex (MHC) and inflammatory disorder risk, two versions of the PRSs were generated; one that included only a single SNP to represent the extended MHC region (chromosome 6: 25-34Mb) and one that omitted all SNPs from the extended MHC region. A single SNP was used in the former set of scores as the MHC has been shown to have extensive patterns of LD.

Cognitive measures

Cognitive testing was carried-out during a clinic visit of children at age 8 years using a short form Wechsler Intelligence Scale for Children (WISC-III).[7] Age adjusted total IQ, performance IQ and verbal IQ measures were derived using the look-up tables provided in the WISC manual. Additional measures included working memory (measured using the backwards digit span task), verbal learning (measured using an adapted version of the nonword repetition test[8]), processing speed (measured using the coding performance subtest) and problem solving (measured using the block design performance subtest). Measures from the Test of Everyday Attention for Children (TEA-Ch)[9] for selective attention (Sky search task) and attentional control (Opposite worlds task) were also investigated. An additional measure of IQ was also captured at age 15 years during a clinic visit using the Wechsler Abbreviated Scale of Intelligence.[10] It has been noted that the WASI is reliable as an estimate of intelligence but tends to underestimates IQ within the ALSPAC cohort. It can therefore not be directly compared to the WISC-III IQ measures. All cognitive measures were standardized to have a mean of zero and a standard deviation (sd) of 1 before analysis.

Psychiatric measures

Anxiety and depression

Presence of any anxiety (generalized anxiety, panic disorder, agoraphobia, social phobia or specific phobia) and ICD-10 diagnoses of depression at age 18 years were measured using the Clinical Interview Schedule-Revised (CIS-R),[11] a self-administered, computerized interview that establishes the severity of anxiety and depression disorder symptoms.

Negative symptoms

Negative symptoms were assessed using 10 questions based on items from the Community Assessment of Psychic Experiences (CAPE) self-report questionnaire[12] at age 16.5 years which measures negative symptoms such as apathy, anergia and asociality. Each item was rated on a 4-point scale (0: never, 1: sometimes, 2: often, 3: always) and a total score was constructed based on the sum of responses (minimum score: 0, maximum score: 30). A binary variable was created using a total score of 14 as a cut-off, chosen to approximately define the top decile (9.18%) of the sample.

Psychotic experiences

The semi-structured Psychosis-Like Symptom Interview (PLIKSi)[13,14] was used to assess psychotic experiences (hallucinations, delusions or experiences of thought interference) at ages 12 and 18 years. To maximize the numbers within our sample, individuals were deemed as having a psychotic experience if rated as having one or more definite psychotic experiences at either age 12 or 18 years, compared to no or only suspected psychotic experiences at age 12 or 18 years.

Attentional deficit/hyperactivity disorder

A parent-reported measure of attentional deficit/hyperactivity disorder was derived from the semi-structured Development and Well Being Assessment (DAWBA) interview at age 7 years, a valid instrument in community and clinical samples.[15] DAWBA bands were generated using a computerized diagnostic algorithm that predicts the likelihood of a clinical diagnostic rating (see http://www.DAWBA.com for more information).[16] We defined individuals as having ADHD if they were categorized in the DAWBA bands predicting a ≥ 15% probability of clinical diagnosis.

Hyperactive and inattentive symptoms

Hyperactive and inattentive symptoms were assessed using the parent-rated 5-item Strengths and Difficulties Questionnaire (SDQ)[17] at approximate ages 4-16 years. At each age individuals were given a hyperactive and inattentive symptoms score ranging from 0-10. Binary measures were created at each age using 7 as a cut-off, as previously used,[18,19] to indicate individuals with abnormal behavior.

Inflammatory markers

When the ALSPAC participants were 9 years of age, blood samples were collected from non-fasting participants and were immediately spun and frozen at -80°C. Inflammatory markers were assayed in 2008 after a median of 7.5 years in storage with no previous freeze-thaw cycles during this period. Interleukin-6 was measured by enzyme-linked immunosorbent assay (R&D Systems, UK), and high-sensitivity C-reactive protein was measured by automated particle-enhanced immunoturbidimetric assay (Roche, UK) as reported in Khandaker *et al.* 2014.[20] All inter- and intra-assay coefficients of variation for interleukin-6 and C-reactive protein were less than 5%.

C-reactive protein was also measured in blood samples were collected from fasting participants who gave consent for venipuncture during clinical assessment at age 16 years. Blood samples were immediately spun, frozen and stored at -80 °C, which were analyzed within 3 - 9 months of blood sampling with no freeze-thaw cycles in between. High sensitivity C-reactive protein was measured by automated particle-enhanced immunoturbidimetric assay (Roche, UK).

Inflammatory marker measures were log transformed before use.

Gene-based analyses

Gene-based analyses were performed using MAGMA (Multi-marker Analysis of GenoMic Annotation).[21] Gene test-statistics were calculated using the "multi" flag option, which aggregates tests using the mean of all SNPs, and the most significant SNP per gene. MAGMA was also used to derive gene-set enrichment statistics. This uses a competitive test to assess whether genes within the gene-set are more associated with the phenotype than genes outside of the gene-set. MAGMA implements this using a specialized version of generalized linear modelling which controls for additional variables including gene-size, linkage disequilibrium and minor allele counts. We tested whether RA associations were enriched in 7 321 gene-ontology gene-sets. To account for multiple testing, we used q-value corrections as implemented in the R Package "qvalue".[22] For gene-sets surviving q-value correction, we used hierarchical clustering analysis to identify gene-sets that were correlated based on shared gene overlap as implemented in the R package "stats".[23] Results of the hierarchical clustering analysis were plotted in as a dendrogram format and broader "cluster" definitions were derived via manual inspection (eFigure 3).

Sensitivity analyses

To assess the robustness of our findings, analyses were repeated using PRSs based on SNPs which were associated with RA, IBD and MS at a range of GWAS *P* value thresholds (P_T; $P \leq 0.5$ to $P \leq 1e^{-7}$) and PRSs that omitted all SNPs from the extended MHC region (chromosome 6: 25-34Mb) (see above). Analyses were also repeated after excluding 9 individuals (0.11% of participants with PRS data) who reported a doctor diagnosis of RA at age 22 years and 649 individuals (8.14% of participants with PRS data)

© 2019 Jones HJ et al. *JAMA Network Open.*

whose mothers had self-reported, when the individuals were 18 years old, that they had ever been told by a doctor that they have arthritis.

Data sources

Summary statistics used to generate polygenic scores are available from the Okada Softwares and Data Source website (http://plaza.umin.ac.jp/~yokada/datasource/software.htm), the International Inflammatory Bowel Disease Genetics Consortium (IIBDGC) website (https://www.ibdgenetics.org/downloads.html) and ImmunoBase (https://www.immunobase.org/).

ALSPAC data used within this study are accessible on request via an online proposal form. Please see http://www.bristol.ac.uk/alspac/researchers/access/ for further details. Please note that the ALSPAC website contains details of all data that are available through a fully searchable data dictionary and variable search tool (http://www.bristol.ac.uk/alspac/researchers/our-data/).

eReferences

1. Boyd A, Golding J, Macleod J, et al. Cohort Profile: the 'children of the 90s' - the index offspring of the Avon Longitudinal Study of Parents and Children. *International Journal of Epidemiology.* 2013;42:111-127.
2. Fraser A, Macdonald-Wallis C, Tilling K, et al. Cohort Profile: the Avon Longitudinal Study of Parents and Children: ALSPAC mothers cohort. *International Journal of Epidemiology.* 2013;42:97-110.
3. Purcell S, Neale B, Todd-Brown K, et al. PLINK: a tool set for whole-genome association and population-based linkage analyses. *American Journal of Human Genetics.* 2007;81:559-575.
4. Okada Y, Wu D, Trynka G, et al. Genetics of rheumatoid arthritis contributes to biology and drug discovery. *Nature.* 2014;506(7488):376-381.
5. Liu JZ, van Sommeren S, Huang H, et al. Association analyses identify 38 susceptibility loci for inflammatory bowel disease and highlight shared genetic risk across populations. *Nature Genetics.* 2015;47(9):979-986.
6. International Multiple Sclerosis Genetics Consortium, Wellcome Trust Case Control Consortium, Sawcer S, et al. Genetic risk and a primary role for cell-mediated immune mechanisms in multiple sclerosis. *Nature.* 2011;476(7359):214-219.
7. Wechsler D. *Wechsler Intelligence Scale for Children, Third UK Edition.* London: The Psychological Corporation; 1992.
8. Gathercole SE, Willis CS, Baddeley AD, Emslie H. The Children's Test of Nonword Repetition: a test of phonological working memory. *Memory.* 1994;2(2):103-127.
9. Manly T, Robertson IH, Anderson V, Nimmo-Smith I. *Test of Everyday Attention for Children (TEA-Ch).* Bury St Edmunds: Thames Valley Test Company; 1998.
10. Wechsler D. *Wechsler Abbreviated Scale of Intelligence.* New York, NY: Psychological Corporation; 1999.
11. Lewis G, Pelosi AJ, Araya R, Dunn G. Measuring Psychiatric-Disorder in the Community - a Standardized Assessment for Use by Lay Interviewers. *Psychol Med.* 1992;22(2):465-486.
12. Stefanis NC, Hanssen M, Smirnis NK, et al. Evidence that three dimensions of psychosis have a distribution in the general population. *Psychol Med.* 2002;32(2):347-358.
13. Horwood J, Salvi G, Thomas K, et al. IQ and non-clinical psychotic symptoms in 12-year-olds: results from the ALSPAC birth cohort. *British Journal of Psychiatry.* 2008;193:185-191.
14. Zammit S, Kounali D, Cannon M, et al. Psychotic experiences and psychotic disorders at age 18 in relation to psychotic experiences at age 12 in a longitudinal population-based cohort study. *Am J Psychiatry.* 2013;170:742-750.
15. Goodman R, Ford T, Richards H, Gatward R, Meltzer H. The Development and Well-Being Assessment: description and initial validation of an integrated assessment of child and adolescent psychopathology. *Journal of Child Psychology and Psychiatry.* 2000;41:645-655.
16. Goodman A, Heiervang E, Collishaw S, Goodman R. The 'DAWBA bands' as an ordered-categorical measure of child mental health: description and validation in British and Norwegian samples. *Soc Psych Psych Epid.* 2011;46(6):521-532.
17. Goodman R. The Strengths and Difficulties Questionnaire: a research note. *Journal of Child Psychology and Psychiatry.* 1997;38(5):581-586.
18. St Pourcain B, Mandy WP, Heron J, Golding J, Smith GD, Skuse DH. Links between co-occurring social-communication and hyperactive-inattentive trait trajectories. *J Am Acad Child Psy.* 2011;50(9):892-902.

© 2019 Jones HJ et al. *JAMA Network Open.*

19. Riglin L, Collishaw S, Thapar AK, et al. Association of genetic risk variants with attention-deficit/hyperactivity disorder trajectories in the general population. *Jama Psychiat.* 2016;73(12):1285-1292.
20. Khandaker GM, Pearson RM, Zammit S, Lewis G, Jones PB. Association of serum interleukin 6 and C-reactive protein in childhood with depression and psychosis in young adult life: a population-based longitudinal study. *Jama Psychiat.* 2014;71(10):1121-1128.
21. de Leeuw CA, Mooij JM, Heskes T, Posthuma D. MAGMA: generalized gene-set analysis of GWAS data. *PLoS Computational Biology.* 2015;11(4):e1004219.
22. Storey JD, Bass AJ, Dabney A, D R. qvalue: Q-value Estimation for False Discovery Rate Control. R Package Version 2.10.0 (http://github.com/jdstorey/qvalue). 2015.
23. R Development Core Team. R: A Language and Environment for Statistical Computing. 2013. http://www.R-project.org/.

eFigures

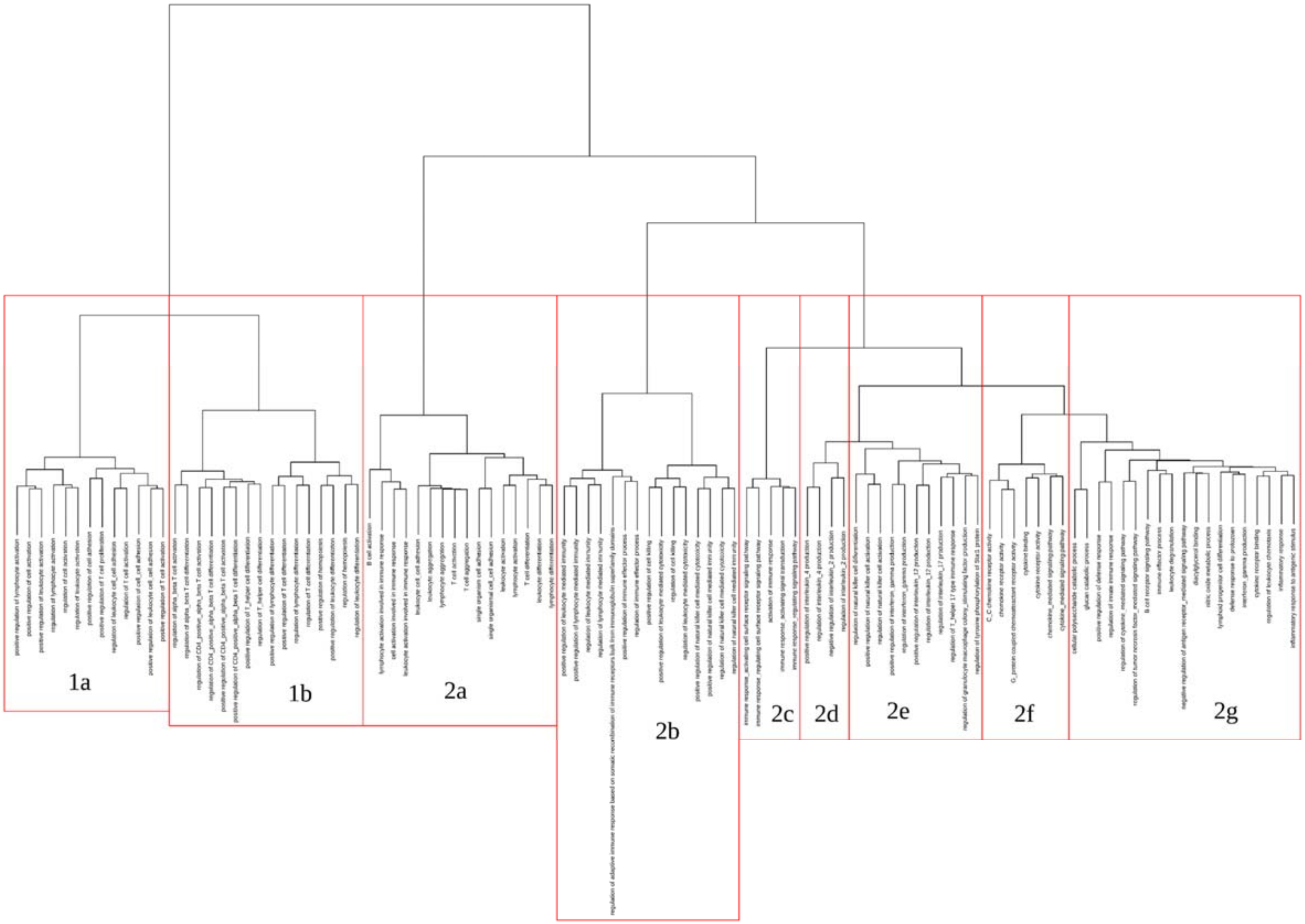

eFigure 1. Identified gene-sets and gene-set sub-clusters. Sub-clusters of cluster 1 (C1): C1a, lymphocyte activation; C1b, lymphocyte differentiation; and SNPs within the gene-set sub-clusters of cluster 2 (C2): C2a, activated lymphocyte homing; C2b, lymphocyte effector functions; C2c, immune activation; C2d, Th2 effector characteristics; C2e, Th1 and Th17 effector characteristics; C2f, lymphokine activities; C2g, immune effector functions. A higher resolution image is available on request from the authors.

© 2019 Jones HJ et al. *JAMA Network Open.*

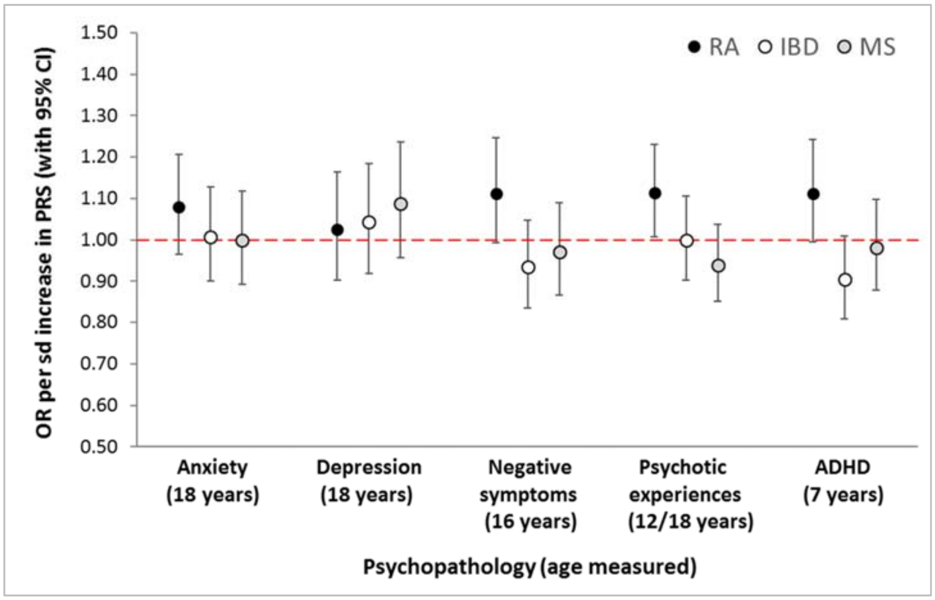

eFigure 2. Associations between polygenic risk scores (PRSs) for rheumatoid arthritis (RA), inflammatory bowel disease (IBD) and multiple sclerosis (MS), and psychopathology measures. Odds ratios (OR) per standard deviation (sd) change in PRS are shown (data markers) with upper and lower error bars indicating 95% confidence intervals (CIs). ORs are shown for PRSs generated using lists of single-nucleotide polymorphisms (SNPs) meeting a 0.05 P value threshold. Red dashed line indicates the null value (1). ADHD, attention deficit/hyperactivity disorder

© 2019 Jones HJ et al. *JAMA Network Open.*

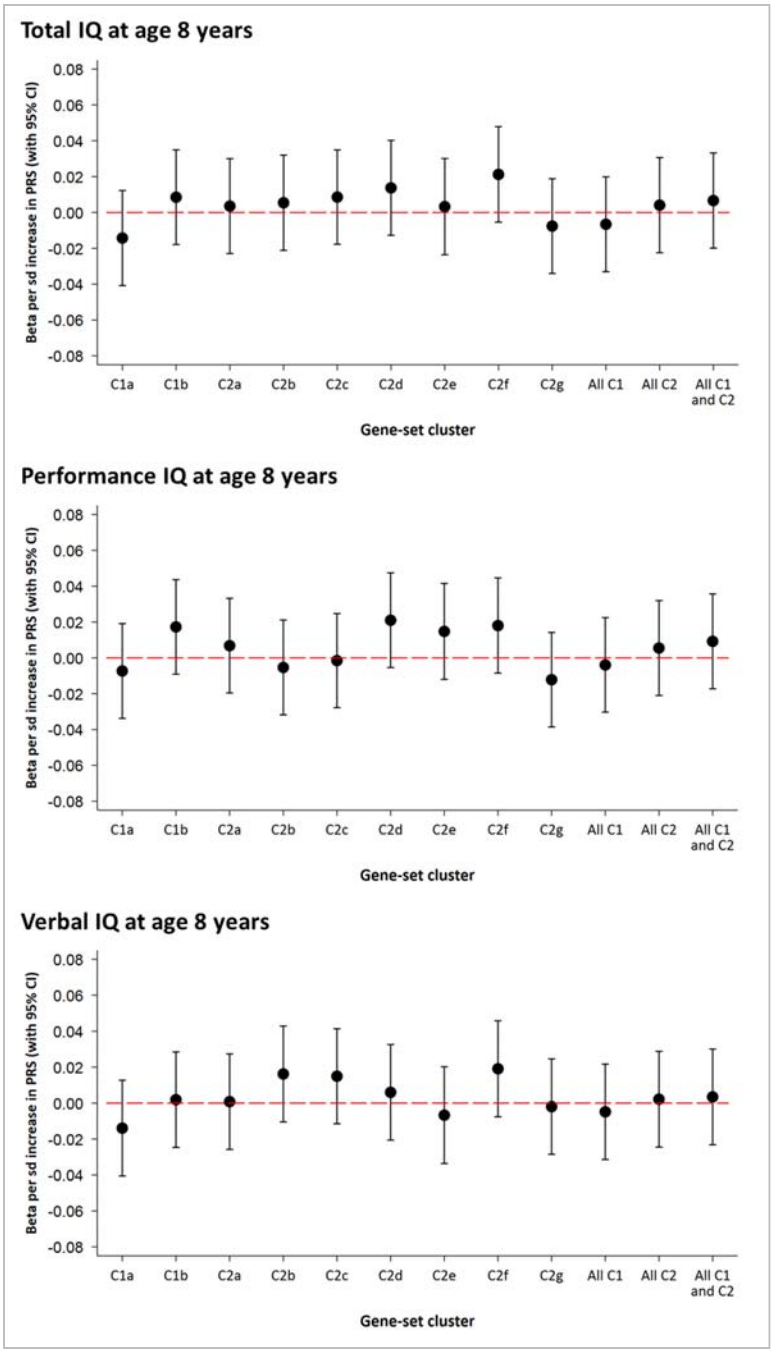

eFigure 3. Associations between polygenic risk scores (PRSs) for rheumatoid arthritis based on single-nucleotide polymorphisms (SNPs) within identified gene-set clusters, and IQ at age 8 years. PRSs were generated using SNPs within the gene-set sub-clusters of cluster 1 (C1): C1a, lymphocyte activation; C1b, lymphocyte differentiation; and SNPs within the gene-set sub-clusters of cluster 2 (C2): C2a, activated lymphocyte homing; C2b, lymphocyte effector functions; C2c, immune activation; C2d, Th2 effector characteristics; C2e, Th1 and Th17 effector characteristics; C2f, lymphokine activities; C2g, immune effector functions. PRSs were also generated using all SNPs within cluster 1 (All C1), cluster 2 (All C2) and clusters 1 and 2 combined (All C1 and C2). Betas per standard deviation (sd) change in PRS are shown (data markers), with upper and lower error bars indicating 95% confidence intervals (CIs). Betas are shown for PRSs generated using lists of SNPs meeting a 0.05 P value threshold. Red dashed line indicates the null value (0).

© 2019 Jones HJ et al. *JAMA Network Open.*

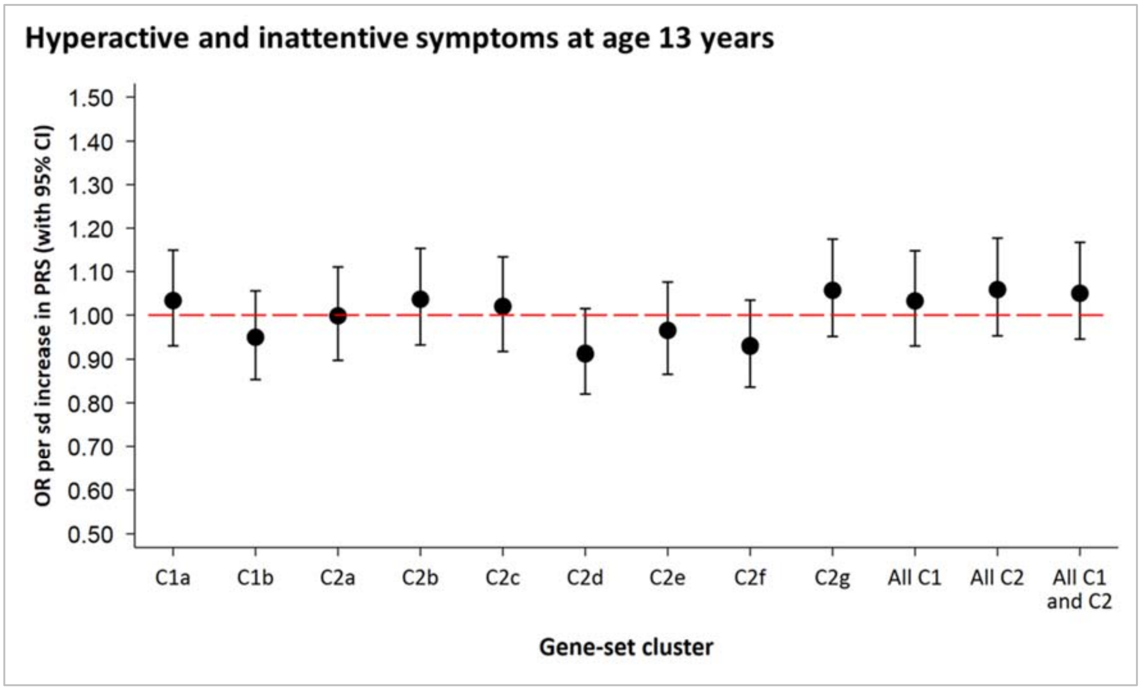

eFigure 4. Associations between polygenic risk scores (PRSs) for rheumatoid arthritis based on single-nucleotide polymorphisms (SNPs) within identified gene-set clusters, and hyperactive and inattentive symptoms at age 13 years. PRSs were generated using SNPs within the gene-set sub-clusters of cluster 1 (C1): C1a, lymphocyte activation; C1b, lymphocyte differentiation; and SNPs within the gene-set sub-clusters of cluster 2 (C2): C2a, activated lymphocyte homing; C2b, lymphocyte effector functions; C2c, immune activation; C2d, Th2 effector characteristics; C2e, Th1 and Th17 effector characteristics; C2f, lymphokine activities; C2g, immune effector functions. PRSs were also generated using all SNPs within cluster 1 (All C1), cluster 2 (All C2) and clusters 1 and 2 combined (All C1 and C2). Odds ratios (OR) per standard deviation (sd) change in PRS are shown (data markers), with upper and lower error bars indicating 95% confidence intervals (CIs). ORs are shown for PRSs generated using lists of SNPs meeting a 0.05 P value threshold. Red dashed line indicates the null value (1).

© 2019 Jones HJ et al. *JAMA Network Open.*

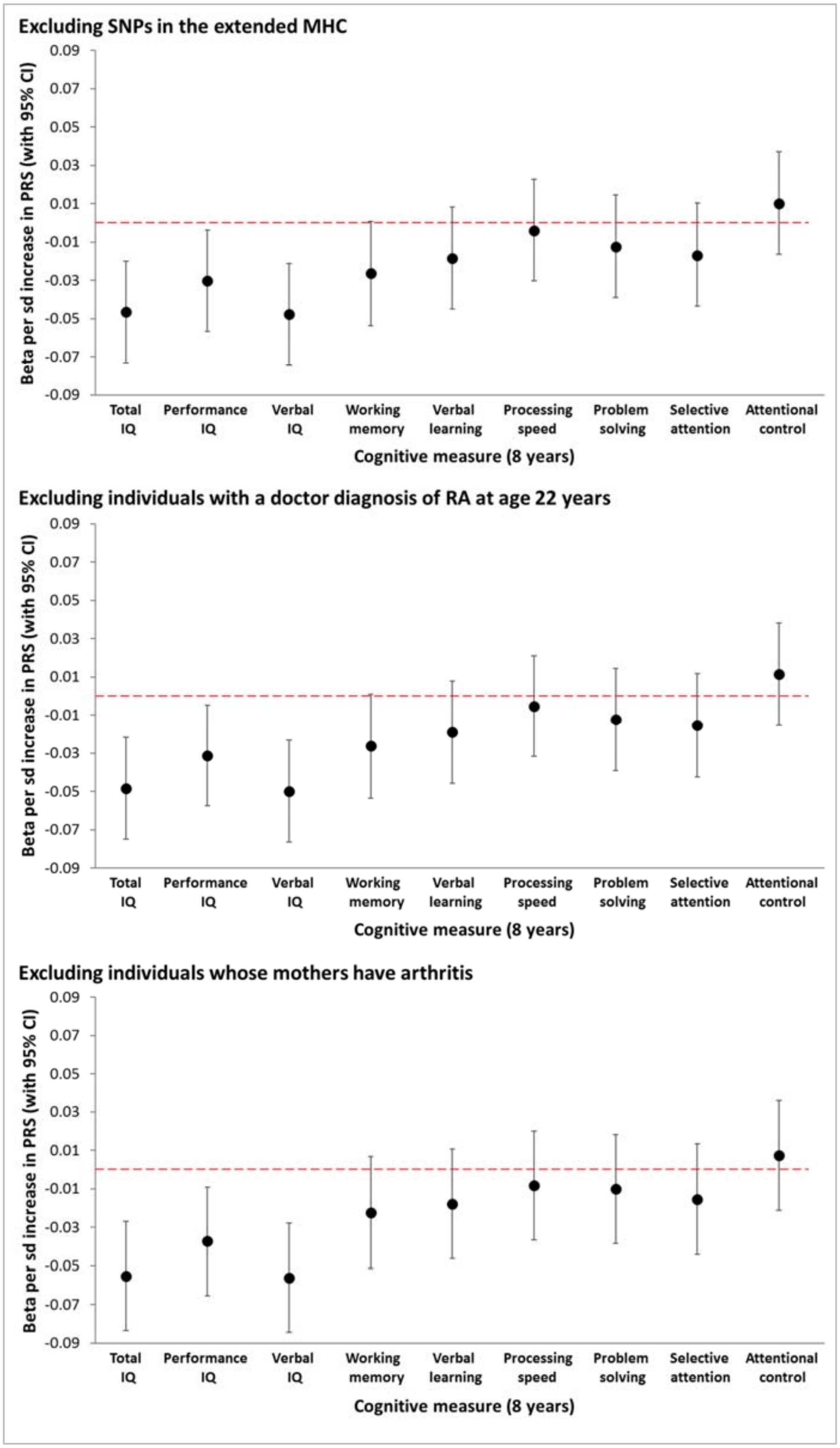

eFigure 5. Sensitivity analysis for associations between rheumatoid arthritis (RA) polygenic risk scores (PRSs) and cognitive phenotypes at age 8 years. Analyses were repeated after excluding all single-nucleotide polymorphisms (SNPs) from the extended major histocompatibility complex (MHC; top figure), after excluding individuals with a doctor diagnosis of RA at age 22 years (middle figure), and after excluding individuals whose mothers had been told by a doctor that they have arthritis (bottom figure). Betas per standard deviation (sd) change in PRS are shown (data markers), with upper and lower error bars indicating 95% confidence intervals (CIs). Betas are shown for PRSs generated using lists of SNPs meeting a 0.05 P value threshold. Red dashed line indicates the null value (0).

© 2019 Jones HJ et al. *JAMA Network Open.*

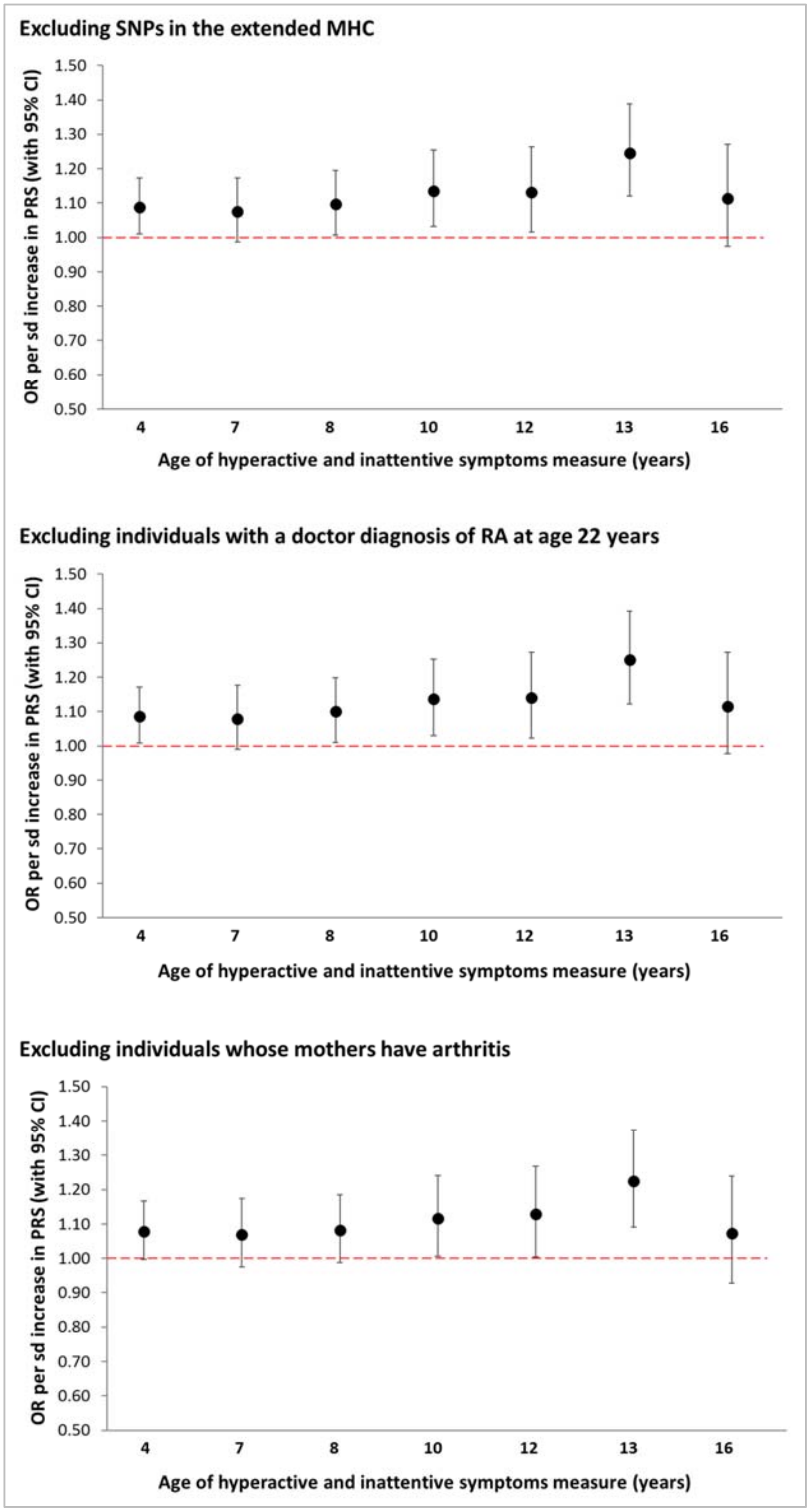

eFigure 6. Sensitivity analysis for associations between rheumatoid arthritis (RA) polygenic risk scores (PRSs) and hyperactive and inattentive symptoms measures at multiple ages. Analyses were repeated after excluding all single-nucleotide polymorphisms (SNPs) from the extended major histocompatibility complex (MHC; top figure), after excluding individuals with a doctor diagnosis of RA at age 22 years (middle figure), and after excluding individuals whose mothers had been told by a doctor that they have arthritis (bottom figure). Odds ratios (OR) per standard deviation (sd) change in PRS are shown (data markers) with upper and lower error bars indicating 95% confidence intervals (CIs). ORs are shown for PRSs generated using lists of SNPs meeting a 0.05 P value threshold. Red dashed line indicates the null value (1).

© 2019 Jones HJ et al. *JAMA Network Open.*

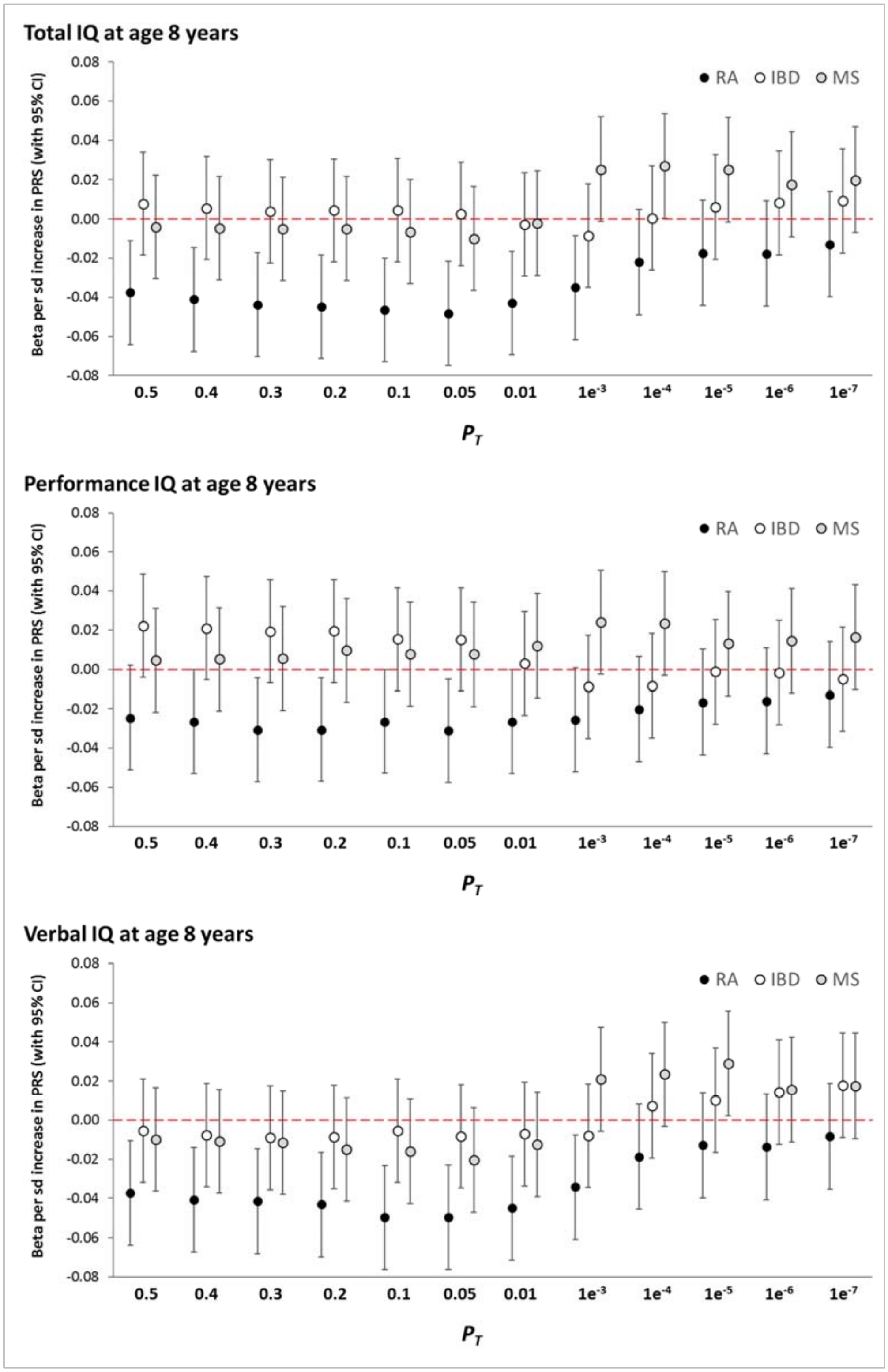

eFigure 7. Associations between polygenic risk scores (PRSs) for rheumatoid arthritis (RA), inflammatory bowel disease (IBD) and multiple sclerosis (MS), and IQ phenotypes at age 8 years. Betas per standard deviation (sd) change in PRS are shown (data markers) for a range of PRSs generated using lists of single-nucleotide polymorphisms meeting a series of P value thresholds (P_T), with upper and lower error bars indicating 95% confidence intervals (CIs). Red dashed line indicates the null value (0).

© 2019 Jones HJ et al. *JAMA Network Open.*

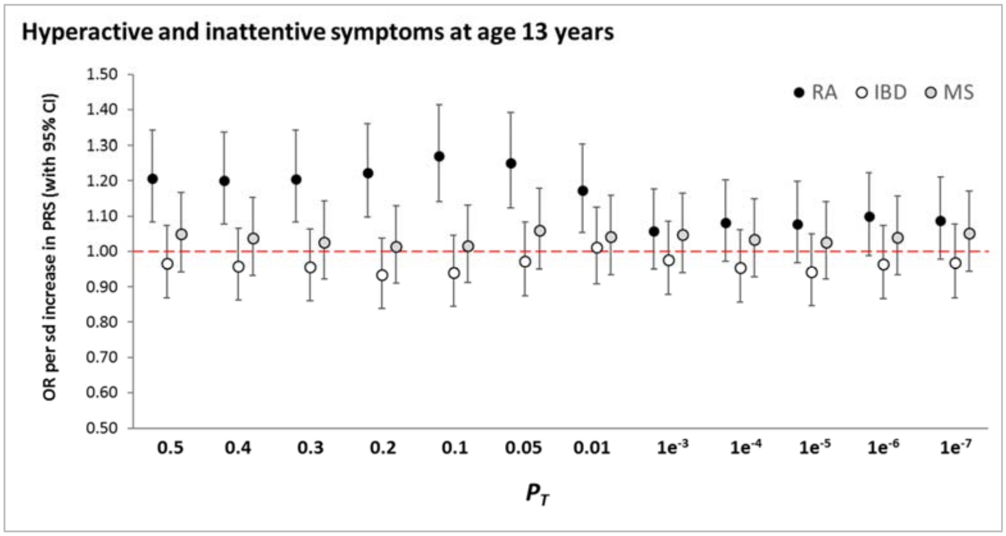

eFigure 8. Associations between polygenic risk scores (PRSs) for rheumatoid arthritis (RA), inflammatory bowel disease (IBD) and multiple sclerosis (MS), and hyperactive and inattentive symptoms at age 13 years. Odds ratios (OR) per standard deviation (sd) change in PRS are shown (data markers) for a range of PRSs generated using lists of single-nucleotide polymorphisms meeting a series of P value thresholds (P_T), with upper and lower error bars indicating 95% confidence intervals (CIs). Red dashed line indicates the null value (1).

© 2019 Jones HJ et al. *JAMA Network Open.*

eTables

eTable 1. Mean age, number of individuals and mean measure for each cognitive outcome measure for the total sample and sample with genetic data.

Measure	Source	Mean age in months (SD)	Total sample		Sample with genetic data	
			N	Mean (SD) of measure[a]	N[b]	Mean (SD) of measure[a]
Total IQ	WISC-III	103.8 (3.9)	7348	104.0 (16.5)	5305	105.1 (16.4)
Performance IQ	WISC-III	103.8 (3.9)	7371	99.5 (17.1)	5320	100.3 (16.9)
Verbal IQ	WISC-III	103.8 (3.9)	7379	107 (16.8)	5328	108.1 (16.7)
Working memory	WISC-III	103.8 (3.9)	7221	3.5 (0.8)	5210	3.5 (0.8)
Verbal learning	WISC-III	103.7 (3.8)	7361	7.2 (2.5)	5334	7.3 (2.5)
Processing speed	WISC-III	103.8 (3.9)	7400	34.5 (7.3)	5340	34.6 (7.2)
Problem solving	WISC-III	103.8 (3.9)	7324	32.2 (12.5)	5282	32.5 (12.5)
Selective attention	TEA-Ch	103.9 (3.9)	7072	5.1 (1.4)	5105	5.1 (1.4)
Attentional control	TEA-Ch	103.9 (3.9)	7156	17.3 (3.7)	5177	17.2 (3.7)

Note: SD, standard deviation; WISC-III, Wechsler Intelligence Scale for Children; TEA-Ch, Test of Everyday Attention for Children.

[a]IQ measures are standard IQ measures as measured by the WISC-III. The working memory score is the raw score (i.e. number of correct answers) from the Backward Digit Span task of the WISC-III. The verbal learning score is the raw score (i.e. number of correct answers) from the Non-word Repetition task of the WISC-III. The processing speed score is the raw score (i.e. number of correct answers) from the Coding task of the WISC-III. The problem solving score is the raw score (i.e. number of correct answers) from the Block Design task of the WISC-III. The selective attention score is measured as the average time taken (in seconds) to identify pairs of identical spaceships (adjusted for motor speed) within the Sky Search task of the TEA-Ch. The attentional control is measured as the average time taken (in seconds) to complete the Opposite Worlds task of the TEA-Ch. All measures were standardised to have a mean of zero and a standard deviation of 1 before analysis.

[b] sample sizes for each association analysis

© 2019 Jones HJ et al. *JAMA Network Open.*

eTable 2. Mean age, number of individuals and proportion of sample with psychopathology for each psychopathology outcome measure for the total sample and sample with genetic data.

Measure	Source	Mean age in months (SD)	Total N (% with psychopathology)	N with genetic data[a] (% with psychopathology)
Hyperactive and inattentive symptoms	SDQ	48.0 (1.5)	9486 (14.3)	5936 (13.3)
		81.4 (1.4)	8400 (11.0)	5531 (10.4)
		98.4 (3.0)	7790 (11.4)	5291 (11.1)
		115.8 (1.5)	8062 (8.1)	5551 (7.8)
		140.6 (1.6)	7331 (7.1)	5129 (6.7)
		157.9 (2.2)	7051 (7.5)	4953 (7.2)
		202.1 (4.3)	5669 (5.5)	4089 (5.6)
ADHD	DAWBA	91.9 (1.7)	8196 (6.1)	5506 (6.0)
Negative symptoms	CAPE	200.1 (2.8)	4976 (9.3)	3519 (9.2)
Anxiety	CIS-R	213.3 (4.6)	4563 (10.2)	3296 (10.1)
Depression	CIS-R	213.3 (4.6)	4563 (7.9)	3296 (7.7)
Psychotic experiences	PLIKSi	153.7 (2.8)/ 213.6 (5.1)[b]	7453 (7.7)	5310 (7.8)

Note: SD, standard deviation; SDQ, Strengths and Difficulties Questionnaire; ADHD, attentional deficit/hyperactivity disorder; DAWBA, Development and Well Being Assessment; CAPE, Community Assessment of Psychic Experiences; CIS-R, Clinical Interview Schedule-Revised; PLIKSi, Psychosis-Like Symptom Interview.

[a] sample sizes for each association analysis

[b] mean age of individuals who completed at least 1 interview session at approximate age 12 and/or 18 years. Individuals were deemed as having a psychotic experience if rated as having one or more definite psychotic experiences at either age.

© 2019 Jones HJ et al. *JAMA Network Open.*

eTable 3. Mean age, number of individuals and mean level of each inflammatory marker measure for the total sample and sample with genetic data.

Measure	Source	Mean age in months (SD)	Total sample		Sample with genetic data	
			N	Median (IQR) of measure	N[a]	Median (IQR) of measure[b]
IL6	Non-fasting blood	118.4 (3.9)	5072	0.8 pg/mL (0.5 - 1.4)	4055	0.8 pg/mL (0.5 - 1.4)
CRP	Non-fasting blood	118.4 (3.9)	5082	0.2 mg/L (0.1 - 0.5)	4064	0.2 mg/L (0.1 - 0.5)
	Fasting blood	185.6 (4.2)	3488	0.4 mg/L (0.2 - 0.9)	2779	0.4 mg/L (0.2 - 0.9)

Note: SD, standard deviation; IL6, Interleukin-6; CRP, C-reactive protein

[a] sample sizes for each association analysis

[b] all measures were log transformed to approximately conform to normality and standardised to have a mean of zero and a standard deviation of 1 before analysis

© 2019 Jones HJ et al. *JAMA Network Open.*

eTable 4. Associations between cognitive measures at age 8 years[a] and polygenic risk scores for inflammatory disorders generated using lists of single-nucleotide polymorphisms meeting a P-value threshold of 0.05.

PRS measure	Measure	N	β (95% CI)	P	R^2
RA	Total IQ	5305	-0.05 (-0.07, -0.02)	<0.001	2.39E-03
	Performance IQ	5320	-0.03 (-0.06, -0.005)	0.021	1.01E-03
	Verbal IQ	5328	-0.05 (-0.08, -0.02)	<0.001	2.51E-03
	Working memory	5210	-0.03 (-0.05, 0.001)	0.058	6.88E-04
	Verbal learning	5334	-0.02 (-0.05, 0.01)	0.174	3.46E-04
	Processing speed	5340	-0.01 (-0.03, 0.02)	0.679	3.21E-05
	Problem solving	5282	-0.01 (-0.04, 0.01)	0.364	1.56E-04
	Selective attention	5105	-0.02 (-0.04, 0.01)	0.260	2.49E-04
	Attentional control	5177	0.01 (-0.02, 0.04)	0.405	1.34E-04
IBD	Total IQ	5305	0.002 (-0.02, 0.03)	0.838	7.85E-06
	Performance IQ	5320	0.02 (-0.01, 0.04)	0.255	2.44E-04
	Verbal IQ	5328	-0.01 (-0.03, 0.02)	0.540	7.05E-05
	Working memory	5210	-0.02 (-0.05, 0.01)	0.191	3.29E-04
	Verbal learning	5334	0.02 (-0.01, 0.04)	0.196	3.14E-04
	Processing speed	5340	-0.01 (-0.03, 0.02)	0.578	5.78E-05
	Problem solving	5282	0.01 (-0.02, 0.03)	0.655	3.78E-05
	Selective attention	5105	-0.002 (-0.03, 0.03)	0.891	3.65E-06
	Attentional control	5177	0.002 (-0.02, 0.03)	0.885	4.05E-06
MS	Total IQ	5305	-0.01 (-0.04, 0.02)	0.460	1.03E-04
	Performance IQ	5320	0.01 (-0.02, 0.03)	0.567	6.16E-05
	Verbal IQ	5328	-0.02 (-0.05, 0.01)	0.135	4.20E-04
	Working memory	5210	0.001 (-0.03, 0.03)	0.959	4.95E-07
	Verbal learning	5334	-0.01 (-0.03, 0.02)	0.638	4.16E-05
	Processing speed	5340	0.01 (-0.02, 0.03)	0.575	5.90E-05
	Problem solving	5282	0.02 (-0.01, 0.05)	0.154	3.85E-04
	Selective attention	5105	0.01 (-0.02, 0.03)	0.668	3.60E-05
	Attentional control	5177	-0.03 (-0.05, 0.002)	0.066	6.55E-04

Note: PRS, polygenic risk score; N, analysis sample size; β, linear regression coefficients representing a standard deviation change in the cognitive measure per standard deviation change in inflammatory disorder PRS; 95% CI, 95% confidence interval; P, linear regression p-value; R^2, proportion of variance in the cognitive measure which can be explained by the inflammatory disorder PRS; RA, rheumatoid arthritis; IBD, inflammatory bowel disease; MS, multiple sclerosis

[a] see eTable 1 for exact ages in months

© 2019 Jones HJ et al. *JAMA Network Open.*

eTable 5. Associations between measures of psychopathology and polygenic risk scores for inflammatory disorders generated using lists of single-nucleotide polymorphisms meeting a P-value threshold of 0.05.

PRS measure	Measure	Age (years)[a]	N	OR (95% CI)	P	pR^2
RA	Hyperactive and inattentive symptoms	4	5936	1.09 (1.01, 1.17)	0.029	1.03E-03
		7	5531	1.08 (0.99, 1.18)	0.084	8.10E-04
		8	5291	1.10 (1.01, 1.20)	0.026	1.34E-03
		10	5551	1.14 (1.03, 1.26)	0.009	2.23E-03
		12	5129	1.14 (1.02, 1.27)	0.018	2.22E-03
		13	4953	1.25 (1.12, 1.39)	<0.001	6.51E-03
		16	4089	1.12 (0.98, 1.27)	0.106	1.48E-03
	ADHD	7	5506	1.11 (0.99, 1.24)	0.062	1.40E-03
	Negative symptoms	16	3519	1.11 (0.99, 1.25)	0.066	1.57E-03
	Anxiety	18	3296	1.08 (0.96, 1.21)	0.185	8.13E-04
	Depression	18	3296	1.03 (0.90, 1.16)	0.698	8.37E-05
	Psychotic experiences	12/18	5310	1.11 (1.01, 1.23)	0.035	1.54E-03
IBD	Hyperactive and inattentive symptoms	4	5936	1.01 (0.94, 1.09)	0.838	8.98E-06
		7	5531	0.90 (0.82, 0.98)	0.013	1.69E-03
		8	5291	0.93 (0.86, 1.02)	0.114	6.78E-04
		10	5551	0.96 (0.87, 1.06)	0.446	1.90E-04
		12	5129	0.96 (0.86, 1.07)	0.412	2.67E-04
		13	4953	0.97 (0.87, 1.08)	0.610	1.01E-04
		16	4089	0.94 (0.82, 1.07)	0.315	5.71E-04
	ADHD	7	5506	0.90 (0.81, 1.01)	0.074	1.28E-03
	Negative symptoms	16	3519	0.93 (0.83, 1.05)	0.242	6.34E-04
	Anxiety	18	3296	1.01 (0.90, 1.13)	0.889	8.97E-06
	Depression	18	3296	1.04 (0.92, 1.18)	0.516	2.36E-04
	Psychotic experiences	12/18	5310	1.00 (0.90, 1.11)	0.989	6.09E-08
MS	Hyperactive and inattentive symptoms	4	5936	0.99 (0.92, 1.06)	0.720	2.77E-05
		7	5531	0.96 (0.88, 1.05)	0.357	2.30E-04
		8	5291	1.01 (0.93, 1.10)	0.799	1.75E-05
		10	5551	1.00 (0.90, 1.10)	0.935	2.17E-06
		12	5129	1.00 (0.90, 1.12)	1.000	4.22E-11
		13	4953	1.06 (0.95, 1.18)	0.299	4.20E-04
		16	4089	0.94 (0.82, 1.08)	0.403	3.97E-04
	ADHD	7	5506	0.98 (0.88, 1.10)	0.746	4.20E-05
	Negative symptoms	16	3519	0.97 (0.87, 1.09)	0.620	1.14E-04
	Anxiety	18	3296	1.00 (0.89, 1.12)	0.978	3.51E-07
	Depression	18	3296	1.09 (0.96, 1.24)	0.198	9.25E-04
	Psychotic experiences	12/18	5310	0.94 (0.85, 1.04)	0.219	5.21E-04

Note: PRS, polygenic risk score; N, analysis sample size; OR, logistic regression odds ratio representing a change in odds of the psychopathology measure compared to baseline per standard deviation change in inflammatory disorder PRS; 95% CI, 95% confidence interval; P, logistic regression p-value; pR^2, pseudo R^2 used to evaluate the goodness-of-fit of logistic models; RA, rheumatoid arthritis; IBD, inflammatory bowel disease; MS, multiple sclerosis; ADHD, attention deficit/hyperactivity disorder

[a] see eTable 2 for exact ages in months

© 2019 Jones HJ et al. *JAMA Network Open.*

eTable 6. Associations between measures of inflammatory markers and polygenic risk scores for inflammatory disorders generated using lists of single-nucleotide polymorphisms meeting a P-value threshold of 0.05.

PRS measure	Measure	Age (years)[a]	N	β (95% CI)	P	R^2
RA	IL6	9	4055	0.004 (-0.03, 0.03)	0.813	1.38E-05
	CRP	9	4064	0.001 (-0.03, 0.03)	0.961	5.84E-07
		16	2779	0.01 (-0.03, 0.04)	0.735	4.11E-05
IBD	IL6	9	4055	0.01 (-0.02, 0.04)	0.560	8.39E-05
	CRP	9	4064	-0.001 (-0.03, 0.03)	0.949	1.00E-06
		16	2779	0.001 (-0.04, 0.04)	0.962	8.12E-07
MS	IL6	9	4055	0.002 (-0.03, 0.03)	0.918	2.61E-06
	CRP	9	4064	0.01 (-0.03, 0.04)	0.735	2.82E-05
		16	2779	0.01 (-0.03, 0.05)	0.597	1.01E-04

Note: PRS, polygenic risk score; N, analysis sample size; β, linear regression coefficients representing a standard deviation change in the inflammatory marker measure per standard deviation change in inflammatory disorder PRS; 95% CI, 95% confidence interval; P, linear regression p-value; R^2, proportion of variance in the inflammatory marker which can be explained by the inflammatory disorder PRS; RA, rheumatoid arthritis; IBD, inflammatory bowel disease; MS, multiple sclerosis; IL6, Interleukin-6; CRP, C-reactive protein

[a] see eTable 3 for exact ages in months

© 2019 Jones HJ et al. *JAMA Network Open.*

eTable 7. Associations between polygenic risk scores for rheumatoid arthritis based on single-nucleotide polymorphisms meeting a P-value threshold of 0.05 within identified gene-set clusters, and IQ at age 8 years[a].

Gene-set cluster PRS[a]	Measure (Age)	N	β (95% CI)	P	R^2
C1a	Total IQ (8years)	5305	-0.01 (-0.04, 0.01)	0.292	2.10E-04
C1b			0.01 (-0.02, 0.03)	0.528	7.52E-05
C2a			0.004 (-0.02, 0.03)	0.792	1.32E-05
C2b			0.01 (-0.02, 0.03)	0.689	3.03E-05
C2c			0.01 (-0.02, 0.03)	0.523	7.71E-05
C2d			0.01 (-0.01, 0.04)	0.309	1.95E-04
C2e			0.003 (-0.02, 0.03)	0.811	1.08E-05
C2f			0.02 (-0.01, 0.05)	0.118	4.62E-04
C2g			-0.01 (-0.03, 0.02)	0.574	5.95E-05
All C1			-0.01 (-0.03, 0.02)	0.626	4.48E-05
All C2			0.004 (-0.02, 0.03)	0.763	1.72E-05
All C1 and C2			0.01 (-0.02, 0.03)	0.623	4.55E-05
C1a	Performance IQ (8years)	5320	-0.01 (-0.03, 0.02)	0.587	5.54E-05
C1b			0.02 (-0.01, 0.04)	0.199	3.10E-04
C2a			0.01 (-0.02, 0.03)	0.613	4.80E-05
C2b			-0.01 (-0.03, 0.02)	0.692	2.95E-05
C2c			-0.002 (-0.03, 0.02)	0.908	2.49E-06
C2d			0.02 (-0.01, 0.05)	0.119	4.57E-04
C2e			0.01 (-0.01, 0.04)	0.279	2.20E-04
C2f			0.02 (-0.01, 0.04)	0.183	3.33E-04
C2g			-0.01 (-0.04, 0.01)	0.365	1.55E-04
All C1			-0.004 (-0.03, 0.02)	0.769	1.62E-05
All C2			0.01 (-0.02, 0.03)	0.684	3.11E-05
All C1 and C2			0.01 (-0.02, 0.04)	0.495	8.77E-05
C1a	Verbal IQ (8years)	5328	-0.01 (-0.04, 0.01)	0.305	1.98E-04
C1b			0.002 (-0.02, 0.03)	0.888	3.74E-06
C2a			0.001 (-0.03, 0.03)	0.952	6.81E-07
C2b			0.02 (-0.01, 0.04)	0.234	2.66E-04
C2c			0.01 (-0.01, 0.04)	0.268	2.31E-04
C2d			0.01 (-0.02, 0.03)	0.658	3.68E-05
C2e			-0.01 (-0.03, 0.02)	0.626	4.47E-05
C2f			0.02 (-0.01, 0.05)	0.161	3.69E-04
C2g			-0.002 (-0.03, 0.02)	0.884	3.97E-06
All C1			-0.005 (-0.03, 0.02)	0.721	2.40E-05
All C2			0.002 (-0.02, 0.03)	0.873	4.78E-06
All C1 and C2			0.003 (-0.02, 0.03)	0.799	1.21E-05

Note: PRS, polygenic risk score; N, analysis sample size; β, linear regression coefficients representing a standard deviation change in IQ per standard deviation change in the rheumatoid arthritis (RA) PRS; 95% CI, 95% confidence interval; P, linear regression p-value; R^2, proportion of variance in IQ which can be explained by the RA PRS

[a] PRSs were generated using single-nucleotide polymorphisms (P-value threshold ≤ 0.05) within the gene-set sub-clusters of cluster 1 (C1): C1a, lymphocyte activation; C1b, lymphocyte differentiation; and SNPs within the gene-set sub-clusters of cluster 2 (C2): C2a, activated lymphocyte homing; C2b, lymphocyte effector functions; C2c, immune activation; C2d, Th2 effector characteristics; C2e, Th1 and Th17 effector characteristics; C2f, lymphokine activities; C2g, immune effector functions. PRSs were also generated using all SNPs within cluster 1 (All C1), cluster 2 (All C2) and clusters 1 and 2 combined (All C1 and C2)

[a] see eTable 1 for exact ages in months

© 2019 Jones HJ et al. *JAMA Network Open.*

eTable 8. Associations between polygenic risk scores for rheumatoid arthritis based on single-nucleotide polymorphisms meeting a P-value threshold of 0.05 within identified gene-set clusters, and hyperactive and inattentive symptoms at age 13 years.

Gene-set cluster PRS[a]	Measure (Age)[a]	N	OR (95% CI)	P	pR^2
C1a	Hyperactive and inattentive symptoms (13 years)	4953	1.03 (0.93, 1.15)	0.544	1.43E-04
C1b			0.95 (0.85, 1.06)	0.334	3.63E-04
C2a			1.00 (0.90, 1.11)	0.973	4.40E-07
C2b			1.04 (0.93, 1.15)	0.512	1.67E-04
C2c			1.02 (0.92, 1.13)	0.713	5.27E-05
C2d			0.91 (0.82, 1.01)	0.091	1.11E-03
C2e			0.96 (0.87, 1.08)	0.519	1.62E-04
C2f			0.93 (0.84, 1.03)	0.180	6.98E-04
C2g			1.06 (0.95, 1.17)	0.305	4.09E-04
All C1			1.03 (0.93, 1.15)	0.553	1.37E-04
All C2			1.06 (0.95, 1.18)	0.292	4.32E-04
All C1 and C2			1.05 (0.94, 1.17)	0.364	3.20E-04

Note: PRS, polygenic risk score; N, analysis sample size; OR, logistic regression odds ratio representing a change in odds of hyperactive and inattentive symptoms compared to baseline per standard deviation change in the rheumatoid arthritis (RA) PRS; 95% CI, 95% confidence interval; P, logistic regression p-value; pR^2, pseudo R^2 used to evaluate the goodness-of-fit of logistic models

[a] PRSs were generated using single-nucleotide polymorphisms (P-value threshold ≤ 0.05) within the gene-set sub-clusters of cluster 1 (C1): C1a, lymphocyte activation; C1b, lymphocyte differentiation; and SNPs within the gene-set sub-clusters of cluster 2 (C2): C2a, activated lymphocyte homing; C2b, lymphocyte effector functions; C2c, immune activation; C2d, Th2 effector characteristics; C2e, Th1 and Th17 effector characteristics; C2f, lymphokine activities; C2g, immune effector functions. PRSs were also generated using all SNPs within cluster 1 (All C1), cluster 2 (All C2) and clusters 1 and 2 combined (All C1 and C2)

[a] see eTable 2 for exact ages in months

© 2019 Jones HJ et al. *JAMA Network Open.*

eTable 9. Sensitivity analysis for associations between rheumatoid arthritis polygenic risk scores and cognitive phenotypes at age 8 years[a].

Sensitivity analysis	Measure	N	β (95% CI)	P	R^2
excluding all SNPs from the extended MHC region	Total IQ	5305	-0.05 (-0.07, -0.02)	0.001	2.23E-03
	Performance IQ	5320	-0.03 (-0.06, 0)	0.025	9.47E-04
	Verbal IQ	5328	-0.05 (-0.07, -0.02)	<0.001	2.33E-03
	Working memory	5210	-0.03 (-0.05, 0)	0.056	7.01E-04
	Verbal learning	5334	-0.02 (-0.05, 0.01)	0.177	3.42E-04
	Processing speed	5340	0 (-0.03, 0.02)	0.774	1.54E-05
	Problem solving	5282	-0.01 (-0.04, 0.01)	0.373	1.50E-04
	Selective attention	5105	-0.02 (-0.04, 0.01)	0.228	2.85E-04
	Attentional control	5177	0.01 (-0.02, 0.04)	0.450	1.10E-04
Excluding individuals with a doctor diagnosis of RA at age 22 years	Total IQ	5299	-0.05 (-0.07, -0.02)	<0.001	2.40E-03
	Performance IQ	5314	-0.03 (-0.06, 0)	0.021	1.01E-03
	Verbal IQ	5322	-0.05 (-0.08, -0.02)	<0.001	2.53E-03
	Working memory	5204	-0.03 (-0.05, 0)	0.058	6.93E-04
	Verbal learning	5329	-0.02 (-0.05, 0.01)	0.162	3.66E-04
	Processing speed	5334	-0.01 (-0.03, 0.02)	0.689	3.00E-05
	Problem solving	5276	-0.01 (-0.04, 0.01)	0.364	1.56E-04
	Selective attention	5100	-0.02 (-0.04, 0.01)	0.268	2.40E-04
	Attentional control	5172	0.01 (-0.02, 0.04)	0.407	1.33E-04
Excluding individuals whose mothers had been told by a doctor that they have arthritis	Total IQ	4748	-0.06 (-0.08, -0.03)	<0.001	3.09E-03
	Performance IQ	4760	-0.04 (-0.07, -0.01)	0.009	1.42E-03
	Verbal IQ	4771	-0.06 (-0.08, -0.03)	<0.001	3.14E-03
	Working memory	4663	-0.02 (-0.05, 0.01)	0.133	4.85E-04
	Verbal learning	4772	-0.02 (-0.05, 0.01)	0.218	3.18E-04
	Processing speed	4778	-0.01 (-0.04, 0.02)	0.562	7.05E-05
	Problem solving	4726	-0.01 (-0.04, 0.02)	0.482	1.05E-04
	Selective attention	4572	-0.02 (-0.04, 0.01)	0.292	2.43E-04
	Attentional control	4634	0.01 (-0.02, 0.04)	0.615	5.46E-05

Note: N, analysis sample size; β, linear regression coefficients representing a standard deviation change in the cognitive measure per standard deviation change in rheumatoid arthritis (RA) polygenic risk score (PRS); 95% CI, 95% confidence interval; P, linear regression p-value; R^2, proportion of variance in the cognitive measure which can be explained by the RA PRS

[a] see eTable 1 for exact ages in months

© 2019 Jones HJ et al. *JAMA Network Open.*

eTable 10. Sensitivity analysis for associations between rheumatoid arthritis polygenic risk scores and hyperactive and inattentive symptoms measures at multiple ages.

Sensitivity analysis	Age at measurement (years)[a]	N	β (95% CI)	P	pR^2
Excluding all SNPs from the extended MHC region	4	5936	1.09 (1.01, 1.17)	0.027	1.05E-03
	7	5531	1.08 (0.99, 1.17)	0.099	7.40E-04
	8	5291	1.1 (1.01, 1.2)	0.033	1.23E-03
	10	5551	1.14 (1.03, 1.25)	0.010	2.20E-03
	12	5129	1.13 (1.02, 1.26)	0.024	2.02E-03
	13	4953	1.25 (1.12, 1.39)	0.000	6.38E-03
	16	4089	1.11 (0.97, 1.27)	0.113	1.43E-03
Excluding individuals with a doctor diagnosis of RA at age 22 years	4	5928	1.09 (1.01, 1.17)	0.029	1.03E-03
	7	5524	1.08 (0.99, 1.18)	0.085	8.10E-04
	8	5284	1.1 (1.01, 1.2)	0.029	1.30E-03
	10	5543	1.14 (1.03, 1.25)	0.010	2.16E-03
	12	5122	1.14 (1.02, 1.27)	0.018	2.22E-03
	13	4945	1.25 (1.12, 1.39)	0.000	6.52E-03
	16	4083	1.12 (0.98, 1.27)	0.107	1.48E-03
Excluding individuals whose mothers had been told by a doctor that they have arthritis	4	5312	1.08 (1, 1.17)	0.061	8.37E-04
	7	4912	1.07 (0.97, 1.17)	0.156	6.23E-04
	8	4697	1.08 (0.99, 1.18)	0.093	8.60E-04
	10	4927	1.12 (1, 1.24)	0.040	1.58E-03
	12	4520	1.13 (1, 1.27)	0.042	1.85E-03
	13	4348	1.22 (1.09, 1.37)	0.001	5.28E-03
	16	3510	1.07 (0.93, 1.24)	0.349	5.86E-04

Note: N, analysis sample size; OR, logistic regression odds ratio representing a change in odds of hyperactive and inattentive symptoms compared to baseline per standard deviation change in the rheumatoid arthritis (RA) PRS; 95% CI, 95% confidence interval; P, linear regression p-value; pR^2, pseudo R^2 used to evaluate the goodness-of-fit of logistic models

[a] see eTable 2 for exact ages in months

© 2019 Jones HJ et al. *JAMA Network Open.*

eTable 11. Associations between polygenic risk scores for rheumatoid arthritis, inflammatory bowel disease and multiple sclerosis, and IQ phenotypes at age 8 years[a] across a range of polygenic risk score P value thresholds.

PRS measure	P_T	Total IQ (N = 5305)		Performance IQ (N = 5320)		Verbal IQ (N = 5328)	
		β (95% CI)	R^2	β (95% CI)	R^2	β (95% CI)	R^2
RA	0.5	-0.04 (-0.06, -0.01)	1.45E-03	-0.02 (-0.05, 0.002)	6.19E-04	-0.04 (-0.06, -0.01)	1.40E-03
	0.4	-0.04 (-0.07, -0.01)	1.72E-03	-0.03 (-0.05, 0.00002)	7.21E-04	-0.04 (-0.07, -0.01)	1.68E-03
	0.3	-0.04 (-0.07, -0.02)	1.97E-03	-0.03 (-0.06, -0.004)	9.81E-04	-0.04 (-0.07, -0.01)	1.75E-03
	0.2	-0.04 (-0.07, -0.02)	2.08E-03	-0.03 (-0.06, -0.004)	9.73E-04	-0.04 (-0.07, -0.02)	1.90E-03
	0.1	-0.05 (-0.07, -0.02)	2.22E-03	-0.03 (-0.05, -0.00005)	7.25E-04	-0.05 (-0.08, -0.02)	2.52E-03
	0.05	-0.05 (-0.07, -0.02)	2.39E-03	-0.03 (-0.06, -0.01)	1.01E-03	-0.05 (-0.08, -0.02)	2.51E-03
	0.01	-0.04 (-0.07, -0.02)	1.91E-03	-0.03 (-0.05, -0.0002)	7.32E-04	-0.05 (-0.07, -0.02)	2.08E-03
	1.00E-03	-0.04 (-0.06, -0.01)	1.26E-03	-0.03 (-0.05, 0.001)	6.84E-04	-0.03 (-0.06, -0.01)	1.19E-03
	1.00E-04	-0.02 (-0.05, 0.005)	4.85E-04	-0.02 (-0.05, 0.01)	4.14E-04	-0.02 (-0.05, 0.01)	3.46E-04
	1.00E-05	-0.02 (-0.04, 0.01)	3.03E-04	-0.02 (-0.04, 0.01)	2.76E-04	-0.01 (-0.04, 0.01)	1.63E-04
	1.00E-06	-0.02 (-0.04, 0.01)	3.10E-04	-0.02 (-0.04, 0.01)	2.54E-04	-0.01 (-0.04, 0.01)	1.84E-04
	1.00E-07	-0.01 (-0.04, 0.01)	1.65E-04	-0.01 (-0.04, 0.01)	1.63E-04	-0.01 (-0.04, 0.02)	6.77E-05
IBD	0.5	0.01 (-0.02, 0.03)	6.42E-05	0.02 (-0.004, 0.05)	5.22E-04	-0.01 (-0.03, 0.02)	3.16E-05
	0.4	0.01 (-0.02, 0.03)	3.27E-05	0.02 (-0.01, 0.05)	4.59E-04	-0.01 (-0.03, 0.02)	6.03E-05
	0.3	0.004 (-0.02, 0.03)	1.63E-05	0.02 (-0.01, 0.05)	3.93E-04	-0.01 (-0.04, 0.02)	8.47E-05
	0.2	0.004 (-0.02, 0.03)	2.01E-05	0.02 (-0.01, 0.05)	3.99E-04	-0.01 (-0.04, 0.02)	7.77E-05
	0.1	0.005 (-0.02, 0.03)	2.18E-05	0.02 (-0.01, 0.04)	2.45E-04	-0.01 (-0.03, 0.02)	3.01E-05
	0.05	0.003 (-0.02, 0.03)	7.85E-06	0.02 (-0.01, 0.04)	2.44E-04	-0.01 (-0.03, 0.02)	7.05E-05
	0.01	-0.003 (-0.03, 0.02)	8.21E-06	0.003 (-0.02, 0.03)	1.01E-05	-0.01 (-0.03, 0.02)	5.17E-05
	1.00E-03	-0.01 (-0.03, 0.02)	7.63E-05	-0.01 (-0.04, 0.02)	8.08E-05	-0.01 (-0.03, 0.02)	6.56E-05
	1.00E-04	0.0004 (-0.03, 0.03)	1.84E-07	-0.01 (-0.03, 0.02)	7.07E-05	0.01 (-0.02, 0.03)	5.39E-05
	1.00E-05	0.01 (-0.02, 0.03)	3.86E-05	-0.001 (-0.03, 0.03)	1.51E-06	0.01 (-0.02, 0.04)	1.05E-04
	1.00E-06	0.01 (-0.02, 0.03)	6.97E-05	-0.002 (-0.03, 0.03)	2.59E-06	0.01 (-0.01, 0.04)	2.09E-04
	1.00E-07	0.01 (-0.02, 0.04)	8.74E-05	-0.005 (-0.03, 0.02)	2.50E-05	0.02 (-0.01, 0.04)	3.22E-04

© 2019 Jones HJ et al. *JAMA Network Open.*

eTable 11 continued

PRS measure	P_T	Total IQ (N = 5305)		Performance IQ (N = 5320)		Verbal IQ (N = 5328)	
		β (95% CI)	R^2	β (95% CI)	R^2	β (95% CI)	R^2
MS	0.5	-0.004 (-0.03, 0.02)	1.68E-05	0.005 (-0.02, 0.03)	2.24E-05	-0.01 (-0.04, 0.02)	9.88E-05
	0.4	-0.005 (-0.03, 0.02)	2.20E-05	0.01 (-0.02, 0.03)	2.84E-05	-0.01 (-0.04, 0.02)	1.22E-04
	0.3	-0.005 (-0.03, 0.02)	2.59E-05	0.01 (-0.02, 0.03)	3.22E-05	-0.01 (-0.04, 0.02)	1.35E-04
	0.2	-0.005 (-0.03, 0.02)	2.44E-05	0.01 (-0.02, 0.04)	9.88E-05	-0.02 (-0.04, 0.01)	2.30E-04
	0.1	-0.01 (-0.03, 0.02)	4.31E-05	0.01 (-0.02, 0.03)	6.19E-05	-0.02 (-0.04, 0.01)	2.58E-04
	0.05	-0.01 (-0.04, 0.02)	1.03E-04	0.01 (-0.02, 0.03)	6.16E-05	-0.02 (-0.05, 0.01)	4.20E-04
	0.01	-0.002 (-0.03, 0.02)	4.35E-06	0.01 (-0.01, 0.04)	1.48E-04	-0.01 (-0.04, 0.01)	1.56E-04
	1.00E-03	0.03 (-0.001, 0.05)	6.56E-04	0.02 (-0.002, 0.05)	5.94E-04	0.02 (-0.01, 0.05)	4.43E-04
	1.00E-04	0.03 (0.0005, 0.05)	7.49E-04	0.02 (-0.003, 0.05)	5.64E-04	0.02 (-0.003, 0.05)	5.55E-04
	1.00E-05	0.03 (-0.002, 0.05)	6.41E-04	0.01 (-0.01, 0.04)	1.77E-04	0.03 (0.002, 0.06)	8.41E-04
	1.00E-06	0.02 (-0.01, 0.04)	3.10E-04	0.01 (-0.01, 0.04)	2.17E-04	0.02 (-0.01, 0.04)	2.41E-04
	1.00E-07	0.02 (-0.01, 0.05)	4.00E-04	0.02 (-0.01, 0.04)	2.68E-04	0.02 (-0.01, 0.04)	3.05E-04

Note: PRS, polygenic risk score; P_T, PRS P value thresholds; N, analysis sample size; β, linear regression coefficients representing a standard deviation change in IQ per standard deviation change in inflammatory disorder PRS; 95% CI, 95% confidence interval; R^2, proportion of variance in IQ which can be explained by the inflammatory disorder PRS; RA, rheumatoid arthritis; IBD, inflammatory bowel disease; MS, multiple sclerosis

[a] see eTable 1 for exact ages in months

© 2019 Jones HJ et al. *JAMA Network Open.*

eTable 12. Associations between polygenic risk scores for rheumatoid arthritis, inflammatory bowel disease and multiple sclerosis, and hyperactive and inattentive symptoms at age 13 years[a] across a range of polygenic risk score P value thresholds.

PRS measure	P_T	Hyperactive and inattentive symptoms at age 13 years (N = 4953)	
		OR (95% CI)	pR^2
RA	0.5	1.21 (1.08, 1.34)	4.60E-03
	0.4	1.20 (1.08, 1.34)	4.34E-03
	0.3	1.21 (1.08, 1.34)	4.54E-03
	0.2	1.22 (1.10, 1.36)	5.28E-03
	0.1	1.27 (1.14, 1.41)	7.48E-03
	0.05	1.25 (1.12, 1.39)	6.51E-03
	0.01	1.17 (1.05, 1.30)	3.36E-03
	1.00E-03	1.06 (0.95, 1.18)	4.09E-04
	1.00E-04	1.08 (0.97, 1.20)	7.90E-04
	1.00E-05	1.08 (0.97, 1.20)	7.29E-04
	1.00E-06	1.10 (0.99, 1.22)	1.16E-03
	1.00E-07	1.09 (0.98, 1.21)	9.31E-04
IBD	0.5	0.97 (0.87, 1.07)	1.64E-04
	0.4	0.96 (0.86, 1.07)	2.36E-04
	0.3	0.96 (0.86, 1.06)	2.58E-04
	0.2	0.93 (0.84, 1.04)	6.22E-04
	0.1	0.94 (0.84, 1.05)	5.12E-04
	0.05	0.97 (0.87, 1.08)	1.01E-04
	0.01	1.01 (0.91, 1.13)	1.61E-05
	1.00E-03	0.98 (0.88, 1.09)	7.56E-05
	1.00E-04	0.95 (0.86, 1.06)	2.96E-04
	1.00E-05	0.94 (0.85, 1.05)	4.65E-04
	1.00E-06	0.96 (0.87, 1.07)	1.66E-04
	1.00E-07	0.97 (0.87, 1.08)	1.42E-04
MS	0.5	1.05 (0.94, 1.17)	2.97E-04
	0.4	1.04 (0.93, 1.15)	1.73E-04
	0.3	1.03 (0.92, 1.14)	8.91E-05
	0.2	1.01 (0.91, 1.13)	2.36E-05
	0.1	1.02 (0.91, 1.13)	3.43E-05
	0.05	1.06 (0.95, 1.18)	4.20E-04
	0.01	1.04 (0.93, 1.16)	2.07E-04
	1.00E-03	1.05 (0.94, 1.17)	2.70E-04
	1.00E-04	1.03 (0.93, 1.15)	1.37E-04
	1.00E-05	1.03 (0.92, 1.14)	8.25E-05
	1.00E-06	1.04 (0.93, 1.16)	1.91E-04
	1.00E-07	1.05 (0.94, 1.17)	3.21E-04

Note: PRS, polygenic risk score; P_T, PRS P value thresholds; N, analysis sample size; OR, logistic regression odds ratio representing a change in odds of hyperactive and inattentive symptoms compared to baseline per standard deviation change in inflammatory disorder PRS; 95% CI, 95% confidence interval; pR^2, pseudo R^2 used to evaluate the goodness-of-fit of logistic models; RA, rheumatoid arthritis; IBD, inflammatory bowel disease; MS, multiple sclerosis

[a] see eTable 2 for exact ages in months

© 2019 Jones HJ et al. *JAMA Network Open.*
